# The Ubiquitin Gene Expression Pattern and Sensitivity to *UBB* and *UBC* Knockdown Differentiate Primary 23132/87 and Metastatic MKN45 Gastric Cancer Cells

**DOI:** 10.3390/ijms21155435

**Published:** 2020-07-30

**Authors:** Emanuele Salvatore Scarpa, Filippo Tasini, Rita Crinelli, Chiara Ceccarini, Mauro Magnani, Marzia Bianchi

**Affiliations:** Department of Biomolecular Sciences, University of Urbino Carlo Bo, 61029 Urbino (PU), Italy; f.tasini3@campus.uniurb.it (F.T.); rita.crinelli@uniurb.it (R.C.); chiara.ceccarini0@gmail.com (C.C.); mauro.magnani@uniurb.it (M.M.); marzia.bianchi@uniurb.it (M.B.)

**Keywords:** gastric cancer, ubiquitin, *UBB*, *UBC*, cell viability

## Abstract

Gastric cancer (GC) is one of the most common and lethal cancers. Alterations in the ubiquitin (Ub) system play key roles in the carcinogenetic process and in metastasis development. Overexpression of transcription factors YY1, HSF1 and SP1, known to regulate Ub gene expression, is a predictor of poor prognosis and shorter survival in several cancers. In this study, we compared a primary (23132/87) and a metastatic (MKN45) GC cell line. We found a statistically significant higher expression of three out of four Ub coding genes, *UBC*, *UBB* and *RPS27A*, in MKN45 compared to 23132/87. However, while the total Ub protein content and the distribution of Ub between the conjugated and free pools were similar in these two GC cell lines, the proteasome activity was higher in MKN45. Ub gene expression was not affected upon *YY1*, *HSF1* or *SP1* small interfering RNA (siRNA) transfection, in both 23132/87 and MKN45 cell lines. Interestingly, the simultaneous knockdown of *UBB* and *UBC* mRNAs reduced the Ub content in both cell lines, but was more critical in the primary GC cell line 23132/87, causing a reduction in cell viability due to apoptosis induction and a decrease in the oncoprotein and metastatization marker β-catenin levels. Our results identify *UBB* and *UBC* as pro-survival genes in primary gastric adenocarcinoma 23132/87 cells.

## 1. Introduction

Ubiquitin (Ub) is a highly conserved 76 amino-acid protein that is covalently conjugated to target proteins by the consecutive actions of three enzymes (E1, E2, E3) in a process known as ubiquitylation [1].

Ub conjugation is an incredibly complex post-translational modification involved in the regulation of many cellular processes such as proteasome-mediated proteolysis, but it also has various non-degradative functions, ranging from signal transduction to transcription, from endocytosis to protein trafficking, from DNA repair to cell survival and proliferation [1,2]. In the cell, Ub is dynamically distributed among distinct pools, which mainly include free or unconjugated Ub, and protein-conjugated Ub consisting of one or more Ub molecules that are peptide-linked to protein substrates [3]. Moreover, free unconjugated polyubiquitin chains also contribute to the total cellular Ub content [4].

In humans, Ub is encoded by four genes: *UBB, UBC, UBA52* and *RPS27A* [5,6,7]. *UBB* and *UBC* encode Ub linear polyproteins formed by three and nine Ub monomers, respectively [5], while *UBA52* and *RPS27A* produce a fusion product where the C-terminus of one Ub molecule is fused to a ribosomal protein [6,7]. These precursors are co- and post-translationally processed in their mature forms by deubiquitinases (DUBs), which selectively cleave Ub monomers from their fusion partners [8].

Both *UBB* and *UBC* were found to be upregulated in several cancers and their high expression levels seemed to be essential to sustain the high proliferation rate of cancer cells and to support their ability to overcome increasing cellular stresses [9,10,11]. Indeed, *UBB* silencing in neuroblastoma, hepatocarcinoma, breast and prostate cancer cells significantly decreased the proliferation rate of all lines tested [9]. Similar results were reported by Tang et al. in lung cancer cells, where *UBC* and *UBB* knockdown inhibits cell growth and weakens radioresistance both in vitro and in vivo [10]. Of note, an upregulation of *UBB* and *UBC* has also been detected in many human cancer specimens, when compared with paired normal adjacent tissues [12].

Despite their recognized role in cell survival and proliferation, little is known about the molecular mechanisms regulating *UBC* and *UBB* gene expression in cancer cells. The *UBC* promoter has long been in the repertoire of promoters currently used to drive exogenous gene expression [13], although its regulatory elements, under basal and stressful conditions, have been only recently characterized [14,15,16].

In particular, it has been demonstrated that the transcription factor (TF) Yin Yang 1 (YY1) has a pivotal role in the regulation of basal *UBC* expression, acting both as a gene-specific transactivator and as a positive regulator of intron splicing [15]. A role for Specificity Protein 1 (SP1) in the transcriptional regulation of *UBC* has also been reported [14,17,18]. By contrast, Heat Shock Factor 1 (HSF1) is the main transcription factor involved in the upregulation of *UBC* gene expression under several stress conditions [16,19,20,21]. In addition, several reports have demonstrated the pro-survival and pro-carcinogenetic role of YY1 [22], SP1 [23] and HSF1 [24] in gastric cancer (GC) development.

Gastric adenocarcinoma is one of the most common malignancies in the world, with a high rate of incidence in many countries [25]. The main clinical classification divides GC into two major histological subtypes: intestinal type GC has higher incidence of blood vessel invasion and liver and lung metastases, whereas diffuse type GC spreads more commonly via the lymphatic system to the pleura and peritoneum [26]. Molecular studies of alterations of single genes have provided evidence that intestinal and diffuse type GC evolve via different genetic pathways, which lead to increased resistance to apoptosis induction, uncontrolled cell proliferation and metastasis development, the latter worsening the prognosis of cancer patients [27,28].

Tian et al. [29] showed, through bioinformatics analyses of microarray data, that *UBB*, *UBC*, *UBA52* and *RPS27A* genes were overexpressed in GC human tissue samples when compared with normal stomach tissues. In addition, the authors demonstrated that *UBA52* and *RPS27A* were overexpressed in the lymph node metastases when compared with primary gastric adenocarcinoma samples, but they did not show any results regarding the different expression levels of *UBB* and *UBC* [29]. Therefore, determining the role of the different Ub genes and of the transcription factors (YY1, HSF1 and SP1) known to be involved in Ub gene expression, both in primary and metastatic GC cells, can pave the way for future studies aimed at identifying new biomarkers involved in the carcinogenetic process that leads to the development of gastric adenocarcinoma.

Our results demonstrate the role of *UBB* and *UBC* as pro-survival genes in primary GC cell line 23132/87 and show that the combined silencing of these two Ub genes in the primary gastric adenocarcinoma cells led to a decrease in their viability, exerted through activation of the extrinsic pathway of apoptosis, and a reduction in levels of the oncoprotein β-catenin, which has a role in overproliferation, migration, invasion of various tumors and also in the epithelial to mesenchymal transition (EMT) process [30].

## 2. Results

### 2.1. Characterization of Ub Expression Profile in Primary 23132/87 and Metastatic MKN45 GC Cells

**Figure 1:**
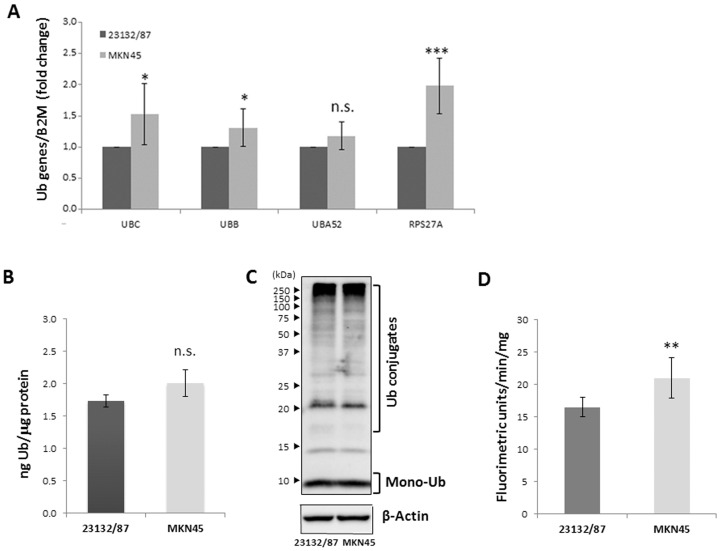
Evaluation of Ub gene expression, Ub protein levels and proteasome activity in 23132/87 and MKN45 cell lines. (**A**) The *UBC*, *UBB*, *UBA52* and *RPS27A* mRNAs were measured by real-time quantitative PCR; the results were normalized to *B2M* mRNA and depicted as fold change compared with the values obtained in 23132/87 cells, set to 1. Data are means ± SD (*n* = 6); * *p* < 0.05, *** *p* < 0.001; n.s. not significant. (**B**) Absolute quantification of total Ub (*n* = 3) using different amounts of Usp2-treated protein extracts and a linear range of Ub standards run in parallel (see Figure S2). Results are given as ng Ub/µg protein; n.s. not significant (*p* = 0.200). (**C**) Representative western immunoblots (*n* = 3) performed using total lysates (5 µg) of 23132/87 and MKN45 cells. The samples were loaded and probed with anti-Ub antibody to detect the poly-Ub and the mono-Ub pools; blots were reprobed with anti-β-Actin antibody, used as a loading control. Brackets indicate Ub conjugates and mono-Ub; arrowheads on the left indicate the molecular weight standards. (**D**) Proteasome activity was assayed in 23132/87 and MKN45 cell extracts using the fluorogenic peptide sLLVY-NH-Mec as a substrate (*n* = 3); the obtained values were expressed as fluorimetric units*min^−1^*mg^−1^; ** *p* < 0.01.

Van der Woude et al. [31] identified the pro-apoptotic protein Fas as a marker of the intestinal type of gastric adenocarcinoma. Our results show that the intestinal type 23132/87 GC cells (Figure S1A) and the hybrid intestinal/diffuse type MKN45 cells (Figure S1B) were both positive for the Fas protein (Figure S1C). Thereafter, we sought to characterize and compare the Ub expression profile in these primary (23132/87) and metastatic (MKN45) GC cell lines. To this end, we evaluated, in both cellular models, the expression levels of the four Ub coding genes, the total cellular Ub content, as well as Ub distribution among the free and conjugated pools and the proteasome activity. When the Ub transcriptome was analyzed, a statistically significant higher expression of three out of the four Ub coding genes, *UBC* (*p* = 0.029), *UBB* (*p* = 0.025) and *RPS27A* (*p* < 0.001), was detected in MKN45 compared to 23132/87 cells, using Beta-2-Microglobulin (*B2M*) for data normalization (Figure 1A). These results were also confirmed using glyceraldehyde 3-phosphate dehydrogenase (*GAPDH*) as a housekeeping gene (Table S1).

While *UBA52* and *RPS27A* are constitutively expressed and contribute with *UBB* and *UBC* to fulfill the ubiquitin demand in basal conditions, only *UBB* and *UBC* are induced when cells are exposed to threats such as heat shock, oxidative stress and proteotoxic stress [19,20,21,32]. To determine total Ub concentration, whole cell extracts from both cell lines were treated with the Usp2 deubiquitinating enzyme to convert all conjugated Ub species into monomeric Ub [33]. Usp2 is able to catalyze the hydrolysis of both Lys-48 and Lys-63 linkages of Ub, thus displaying broad specificity [34]. The total Ub protein content was accurately determined by running different amounts of Usp2-digested protein extracts and a proper range of Ub standards in the same immunoblot (Figure S2). The average of three independent assays indicates a similar (*p* = 0.200) Ub content in 23132/87 and MKN45 cells (1.74 ± 0.10 and 2.01 ± 0.21 ng Ub/µg protein, respectively; Figure 1B), despite the lower *UBC*, *UBB* and *RPS27A* transcript levels in primary GC cells (Figure 1A). Moreover, the Ub distribution between the conjugated and free pools was investigated: MKN45 and 23132/87 cells showed similar distribution of Ub between the two pools (Figure 1C). Since Ub has a pivotal role in proteasome-mediated degradation [35] and in turn the proteasome is responsible for Ub degradation, thus affecting its cellular levels [36], we then determined the proteasome activity of these two GC cell lines by evaluating the chymotrypsin-like activity (that is, the predominant activity) of the 20S core. Our results show that MKN45 cells possess a significantly higher (*p* = 0.009) proteasome activity with respect to 23132/87 cells (Figure 1D).

### 2.2. Cytosolic and Nuclear Distribution of YY1, HSF1 and SP1 in 23132/87 and MKN45 GC Cell Lines

**Figure 2:**
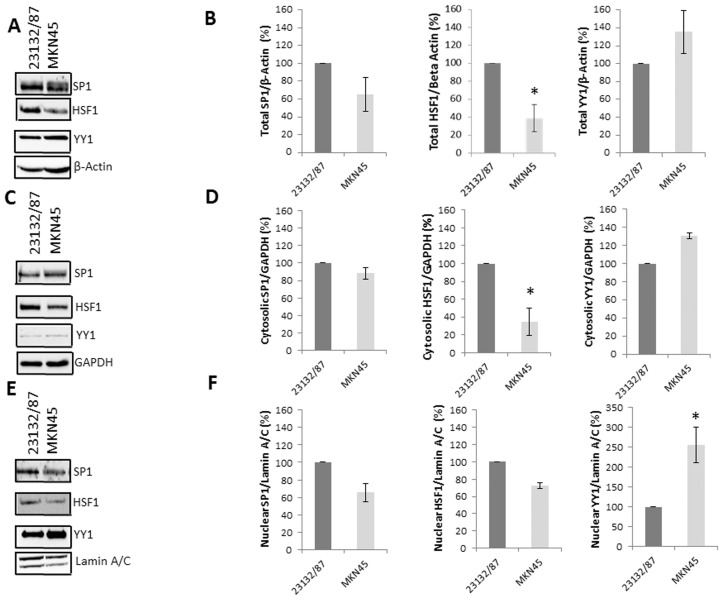
Analysis of total, cytosolic and nuclear levels of transcription factors SP1, HSF1 and YY1 in 23132/87 and MKN45 cell lines. Representative western immunoblots (*n* = 3) performed using total (**A**), cytosolic (**C**) and nuclear (**E**) lysates, which were obtained from 23132/87 and MKN45 cells. Quantities of 15 µg of total or cytosolic cellular proteins and 7.5 µg of nuclear proteins were loaded and probed with antibodies specific for SP1, HSF1 and YY1. Blots were reprobed with antibodies against proteins used as a loading control: anti-β-Actin for total extracts, anti-GAPDH for cytosolic extracts and anti-Lamin A/C for nuclear extracts. Quantification (**B**,**D**,**F**) of SP1, HSF1 and YY1 levels in 23132/87 and MKN45 samples was performed with Image Lab analysis software version 5.2.1. The values obtained from the ratio between SP1, HSF1 and YY1 signals and the loading control β-Actin (**B**) or GAPDH (**D**) or Lamin A/C (**F**) in MKN45 cells were calculated and compared with the values obtained in 23132/87 cells, set as 100%; * *p* < 0.05.

Given the role of YY1 [15,22], HSF1 [16,24] and SP1 [14,17,18,23] both as transcription factors involved in Ub gene expression and as prognostic markers in GC development, the basal levels of these protein factors in whole (Figure 2A,B), cytosolic (Figure 2C,D) and nuclear extracts (Figure 2E,F) of 23132/87 and MKN45 cells were evaluated. Our results show that YY1 total levels were higher in MKN45 compared to 23132/87, while both HSF1 and SP1 levels were lower (Figure 2A,B). The same pattern was generally reflected in the cytosolic (Figure 2C,D) and nuclear content (Figure 2E,F) of these transcription factors. Even if the total and cytosolic HSF1 levels were higher (*p* = 0.049 and *p* = 0.019, respectively) in 23132/87 than in MKN45 cells (Figure 2A,D), both GC cells showed similar HSF1 levels in their nuclear extracts (Figure 2E,F). Meanwhile, the levels of YY1, which is mainly a nuclear factor, were significantly higher (*p* = 0.035) in the nuclei of MKN45 cells (Figure 2E,F). Regarding the SP1 transcription factor, the appreciable, albeit not significant, lower expression in MKN45 versus 23132/87 detected in whole cell lysates was reconfirmed in the nuclear extracts (Figure 2E,F).

### 2.3. Effect of YY1, HSF1 and SP1 Transcription Factor Silencing on Ub Gene Expression

**Figure 3:**
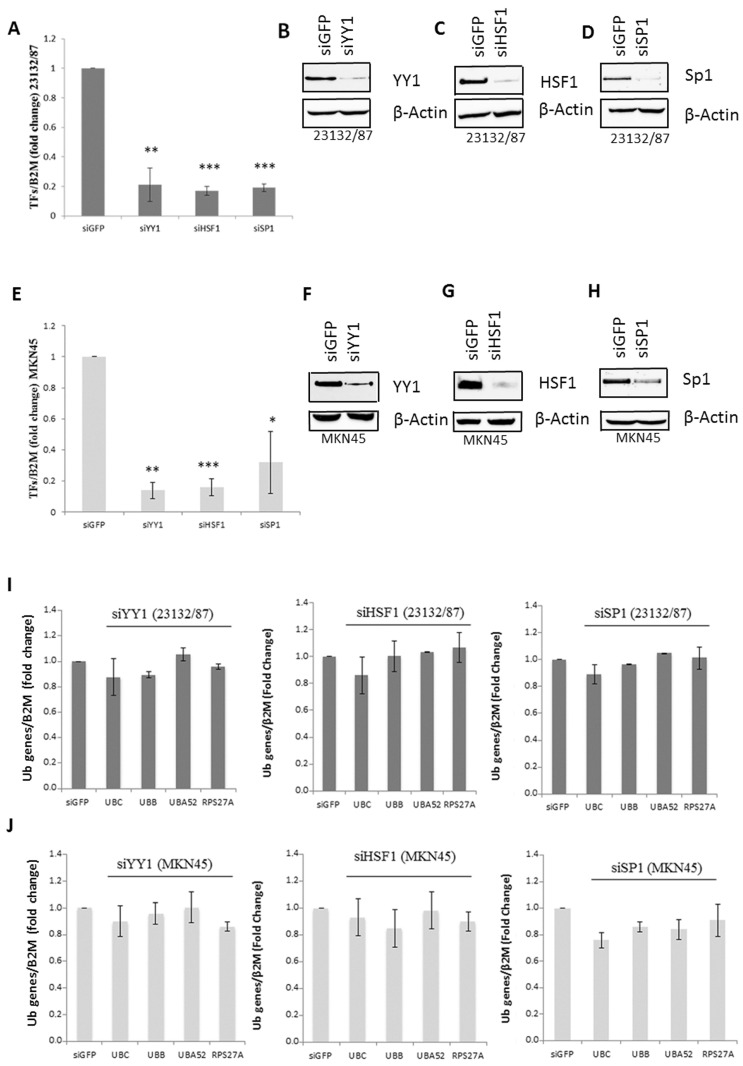
siRNA-mediated knockdown of YY1, HSF1 and SP1 transcription factors and Ub gene expression levels after RNA interference treatments in the 23132/87 and MKN45 cell lines. 23132/87 (**A**) and MKN45 (**E**) cells were transiently transfected with *YY1*, *HSF1*, *SP1* and control *GFP* siRNAs. At 48 h after transfection, the mRNA levels of the target genes were measured by real-time quantitative PCR, normalized to *B2M* mRNA and depicted as fold change compared with si*GFP* transfected cells. Data are means ± SD (*n* = 3); * *p* < 0.05, ** *p* < 0.01, *** *p* < 0.001. Representative western immunoblots of total proteins from 23132/87 (**B**–**D**) and MKN45 (**F**–**H**) cells transfected with either control (si*GFP*) or *YY1*, *HSF1* or *SP1* siRNAs, at 48 h post-transfection (*n* = 3). A quantity of 15 µg of total cellular proteins was loaded and probed with antibodies specific for YY1 (**B**,**F**), HSF1 (**C**,**G**) or SP1 (**D**,**H**); blots were reprobed with anti-β-Actin, used as a loading control. 23132/87 (**I**) and MKN45 (**J**) cells were transiently transfected with *YY1*, *HSF1*, *SP1* and control *GFP* siRNAs. After 48 h, the mRNA levels of *UBC*, *UBB*, *UBA52* and *RPS27A* genes were measured by real-time quantitative PCR, normalized to *B2M* mRNA and depicted as fold change compared with si*GFP* transfected cells, set as 1. Data are means ± SD (*n* = 3).

To investigate the role of YY1, HSF1 and SP1 in determining Ub expression levels in 23132/87 and MKN45 cells, RNA interference experiments targeting these transcription factors were performed. Both cell lines were transfected with siRNAs against either *YY1*, *HSF1* or *SP1* and green fluorescent protein (*GFP*), used as a negative control. Target knockdown was effective at both the mRNA and protein levels (Figure 3A–H). RTqPCR revealed about an 80% decrease in the target mRNA for all three transcription factors in 23132/87 (Figure 3A); MKN45 displayed an average 85% reduction in *HSF1* and *YY1* mRNA levels, and a 68% reduction in the *SP1* mRNA levels (Figure 3E). A consistent reduction was also obtained at the protein level for all three transcription factors in both cell lines (Figure 3B–D and Figure 3F–H). However, TF knockdown did not affect Ub gene transcription, as detected by RTqPCR of cDNA samples derived from silenced GC cells compared to si*GFP* transfected cells (Figure 3I,J). Regarding HSF1, its activity is mainly induced in response to different stressful conditions such as heat shock, oxidative stress or proteotoxic stress [19,21]: this may account for the lack of effect on *UBC* and *UBB* gene transcription in *HSF1* silenced gastric cancer cells. Instead, the output of YY1 transcription factor silencing was further investigated by RTqPCR analysis of three known direct targets of YY1: Bcl-2-like protein 4 (*BAX*) and Avian myelocitomatosis virus oncogene cellular homolog (*c-MYC*), which are positively regulated by YY1 [37], and the survivin coding gene, which is repressed by YY1 [38]. While *BAX* and survivin transcript levels were both unchanged upon *YY1* silencing, *c-MYC* expression showed a significant reduction in both cell lines (28% and 52% reduction in 23132/87 and MKN45, respectively; Supplementary Table S2).

### 2.4. Role of UBB and UBC in Gastric Adenocarcinoma Cell Proliferation and Survival

**Figure 4:**
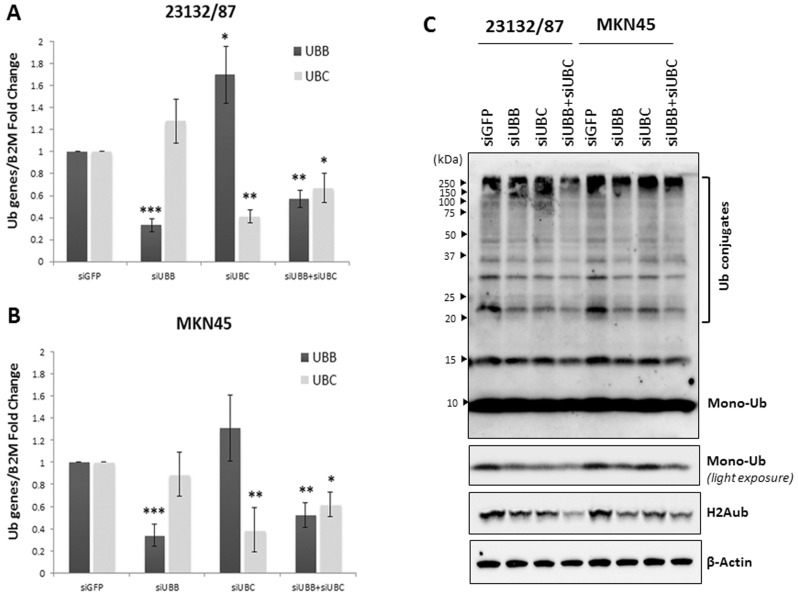
siRNA-mediated knockdown of *UBB* and *UBC* in 23132/87 and MKN45 cell lines. 23132/87 and MKN45 cells were transiently transfected with either control *GFP* or *UBB*, *UBC* or *UBB*+*UBC* siRNAs. At 48 h after transfection, the mRNA levels of *UBB* and *UBC* for 23132/87 (**A**) and MKN45 (**B**) were measured by real-time quantitative PCR, normalized to *B2M* mRNA and depicted as fold change compared with si*GFP* transfected cells, set as 1. Data are means ± SD (*n* = 3); * *p* < 0.05, ** *p* < 0.01, *** *p* < 0.001. (**C**) Representative western immunoblots (*n* = 2) of total protein lysates (5 μg) of 23132/87 and MKN45 cells obtained 48 h after transfection with si*GFP* (control), si*UBB*, si*UBC* and si*UBB*+si*UBC*. The samples were loaded and probed with anti-Ub antibody to detect the conjugated and mono-Ub pools (the latter were analyzed using the light exposure image). Blots were reprobed with anti-H2Aub and anti-β-Actin antibody, used as a loading control. Brackets indicate Ub conjugates. Arrowheads on the left indicate the molecular weight standards.

Tang et al. demonstrated that siRNA-mediated knockdown of *UBB* and *UBC* mRNAs, targeted individually and in combination, led to a reduction in Ub levels in A549 lung cancer cells [10]. In addition, downmodulation of these “pro-survival factors” inhibited cancer cell proliferation, induced the apoptotic process and decreased the tumor size of A549-derived xenografts in vivo [10]. On these bases, we targeted *UBB* and *UBC* in 23132/87 and MKN45 GC cells. siRNA-mediated silencing of *UBB* and *UBC*, alone or in combination, led to a marked decrease in their respective mRNAs (*p* < 0.001 for *UBB* silencing with si*UBB* in both cell lines; *p* = 0.002 and *p* = 0.003 for *UBC* silencing with si*UBC* in 23132/87 and MKN45, respectively; *p* = 0.007 for *UBB* and *p* = 0.029 for *UBC* silencing with si*UBB*+si*UBC* in 23132/87; *p* = 0.009 for *UBB* and *p* = 0.013 for *UBC* silencing with si*UBB*+si*UBC* in MKN45), with *UBB* knockdown being more effective than *UBC* knockdown in both GC cell lines (Figure 4A,B). To ascertain if the downregulation of one poly-Ub gene could be compensated by the transcriptional induction of the other, we evaluated *UBC* and *UBB* transcript levels in si*UBB* and si*UBC* transfected cells, respectively. Data obtained demonstrate that an effective knockdown of *UBC* triggers *UBB* upregulation only in the 23132/87 primary cells (*p* = 0.032), while *UBB* silencing is not compensated for by *UBC* upregulation in the investigated GC cell lines (Figure 4A,B). We then sought to investigate the effects of the siRNA-mediated knockdown of *UBB* and *UBC* on the free and conjugated Ub pools in 23132/87 and MKN45 cells. Our results show that si*UBB*, si*UBC* and si*UBB*+si*UBC* transfections efficiently decreased the conjugated Ub pool in both 23132/87 and MKN45 cell lines, with the most prominent reduction detected in *UBB*+*UBC* silenced GC cells. Moreover, the knockdown of polyubiquitin genes, singularly or in combination, also caused a significant decrease in free Ub levels, with the highest effect observed in the 23132/87 primary cells, transfected with si*UBB*+si*UBC* (Figure 4C). The free Ub content upon siRNA treatments was accurately determined by running whole cell extracts along with a proper range of Ub standards in the same immunoblot (Table S3). Results of two independent experiments indicate a 50.4% reduction in free Ub content in the double knockdown 23132/87 primary cell line, while a similar lower decrease was caused by the single knockdown of *UBB* or *UBC* (Table S3). The MKN45 metastatic cell line underwent a reduction in free Ub levels following *UBB* and *UBC* silencing, with the first one being far more effective (40.5% and 22.5% reduction, respectively). Simultaneous knockdown of both *UBB* and *UBC* genes had no further impact on the free Ub pool compared to *UBB* silencing only (Figure 4C and Table S3). Of note, a reduced level of the immunoreactive band corresponding to ubiquitinated H2A histone is appreciable in both 23132/87 and MKN45 cells treated with *UBB*- and/or *UBC*-targeting siRNAs (Figure 4C).

**Figure 5:**
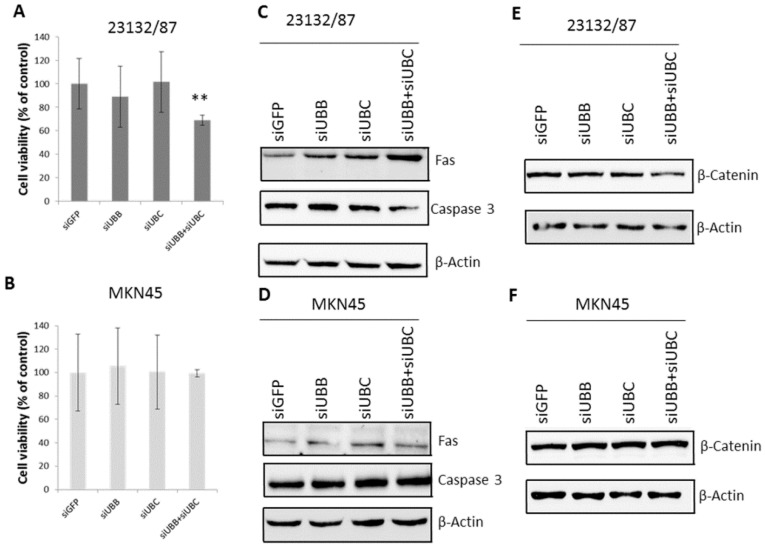
MTS (3-(4,5-dimethylthia-zol-2yl)-5-(3-carboxymethoxyphenyl)-2-(4-sulfophenyl)2*H*- tetrazolium) assay for 23132/87 and MKN45 cells and evaluation of apoptotic and pro-survival markers. Cell viability of 23132/87 (**A**) and MKN45 (**B**) cells transiently transfected (48 h) with either si*GFP* (control) or with *UBB*, *UBC* or *UBB*+*UBC* siRNAs was evaluated via MTS assay (*n* = 8) and shown as a percentage with respect to the si*GFP* transfected cells, set as 100%; ** *p* <0.01. (**C**,**D**) Representative western immunoblots of total proteins from 23132/87 (**C**) or MKN45 (**D**) cells transfected as in (**A**,**B**), respectively (*n* = 3). A quantity of 15 µg of total cellular proteins was loaded and probed with antibodies specific for Fas or Caspase 3; blots were reprobed with anti-β-Actin, used as a loading control. (**E**,**F**) Representative western immunoblots of total proteins from 23132/87 (**E**) or MKN45 (**F**) cells transfected as in (**A**,**B**), respectively (*n* = 2). A quantity of 7.5 µg of total cellular proteins was loaded and probed with the antibody specific for β-catenin; blots were reprobed with anti-β-Actin, used as a loading control.

We then evaluated the pro-survival role of the poly-Ub coding genes in both primary and metastatic GC cell lines. Our results show that the combined silencing of *UBB* and *UBC* genes led to a significant decrease (*p* = 0.004) in 23132/87 cell viability (Figure 5A), detected at 48 h post-transfection, but had no effect on MKN45 cells (Figure 5B). These data indicate that 23132/87 primary GC cells are more strictly dependent on the Ub levels in order to maintain their over-proliferation rate. Finally, we looked for a molecular mechanism accounting for the reduced cell viability observed in the 23132/87 cells after the simultaneous silencing of poly-Ub genes *UBC* and *UBB*. Considering the role of the extrinsic pathway of apoptosis in gastric adenocarcinoma [39], we evaluated if the significant decrease (*p* = 0.004) in 23132/87 cell viability after treatment with the combination si*UBB*+si*UBC* could be linked to an increase in Fas levels [31] and activation of caspase 3. As shown in Figure 5C, si*UBB*+si*UBC* transfection in the 23132/87 cells led to a remarkable increase in the pro-apoptotic protein Fas and to a decrease in the inactive full-length caspase 3, indicating activation of the apoptotic process [28]. Instead, the si*UBB*+si*UBC* treatment did not modify Fas and full-length caspase 3 levels in MKN45 cells (Figure 5D), which is consistent with the lack of effect on cell viability (Figure 5B).

Zhang et al. [40] showed that the transcription factor β-catenin is an oncoprotein in gastric cancer and the Kruppel-like factor 4 (KLF4)-mediated inhibition of β-catenin expression led to a reduction in the proliferation rate, migration and invasion capacities of MKN45 gastric cancer cells. In light of these results, we evaluated the β-catenin protein levels in 23132/87 and MKN45 cancer cells after si*UBB*, si*UBC* and si*UBB*+si*UBC* treatments. *UBB* and *UBC* silencing decreases the β-catenin levels in 23132/87 cells, while in MKN45 cells the β-catenin levels are not affected (Figure 5E,F).

## 3. Discussion

Gastric adenocarcinoma is the second most common cause of cancer-related death worldwide [28]. In a recent study by Tian et al. [29], the four Ub coding genes (*UBA52*, *RPS27A*, *UBB, UBC*) were all identified as gastric-related therapeutic indicators, based on ego network analysis. In fact, their overexpression was found to be related to the progression and metastasis development of gastric cancer [29].

Our results show that Ub gene expression is significantly different in the primary and metastatic GC cell lines investigated, i.e., 23132/87 and MKN45. In particular, the metastatic MKN45 cells display higher levels of *UBB, UBC* and *RPS27A* gene transcription, in agreement with the evidence of Tian et al. [29], which links the upregulation of Ub genes to metastasis development. *UBB* and *UBC* upregulation in several cancers is widely documented [9,10]. Regarding *UBA52*, Zhou et al. [41] showed that, in colorectal cancer, the long non-coding RNA LUCAT1 controls *UBA52* by negatively affecting the Ub-RPL40 protein stability. Since LUCAT1 is also highly expressed in gastric cancer [42], it can be hypothesized that a similar post-translational control also occurs in 23132/87 and MKN45 gastric cancer cells.

In contrast to the transcript levels of the Ub genes, the two GC cell lines 23132/87 and MKN45 showed no significant differences in their total Ub levels and in the Ub distribution between the free and conjugated pools, although the MKN45 GC cell line showed a greater transcription of three out of four Ub coding genes. We hypothesize that different post-transcriptional regulatory mechanisms may account for this discrepancy. In their review, Liu et al. [43] conclude that transcription levels by themselves are not sufficient to predict protein levels in many scenarios in the cell. Multiple processes beyond transcript concentration contribute to establishing the real expression level of a protein: translation rates modulated by non-coding RNAs (such as miRNAs and long non-coding RNAs); protein synthesis delay, in which transcript changes will affect protein levels only with a certain temporal delay; and also modulation of the protein half-life through both ubiquitin-proteasome and lysosomes-autophagy pathways [43]. In addition, Schwanhausser et al. [44] finely evaluated, on a genomic scale, the correlation between mRNA and protein levels and found it to be quite low (R^2^ around 0.4). Furthermore, we demonstrated that the MKN45 cell line has a higher chimotrypsin-like proteasome activity, which could be at least partially responsible for the lack of differences of total Ub levels in 23132/87 and MKN45 cells. In fact, it has been demonstrated that Ub can be degraded by the proteasome following three routes: along with its conjugated substrate, when extended with a C-terminal tail and as a monomer under different pathophysiological conditions [45].

To shed light on the molecular players supporting Ub gene expression in GC cells, we preliminarily evaluated the levels and the intracellular distribution of HSF1, YY1 and SP1 transcription factors in the MKN45 compared to the 23132/87 cell line. Several pieces of evidence in the literature, including our previous studies, indeed reported the involvement of these transcriptional regulators in the expression of the Ub genes, either in basal or stressful conditions [14,15,16,17,18,19,20,21].

Moreover, these transcription factors have all been linked to gastric cancer development [22,23,24]. In particular, *HSF1* knockdown reduced the proliferation, migration and invasiveness capabilities of AGS and MKN28 gastric adenocarcinoma cell lines [24]. *YY1* overexpression enhanced GC cell proliferation, monolayer colony formation and xenograft growth, whereas *YY1* knockdown inhibited GC cell proliferation both in vitro and in vivo [22]. Arora et al. [46] showed that the cytotoxic effect of the drug triptolide in MKN45 and MKN28 GC cells is mediated by a decrease in the SP1 levels and the activation of caspases 3 and 7.

Our results show lower total and cytosolic HSF1 levels in MKN45 cells with respect to 23132/87 cells, although the nuclear levels of the transcription factor are similar in the two cell lines. HSF1 is a key player in the transcriptional programs mounted in stressed cells to maintain proteostasis, which is accomplished by HSF1-mediated expression of stress-protective genes. Interestingly, Gencer and Irmak Yazicioglu [47] demonstrated that 23132/87 cells are more prone to cellular stresses, in particular to oxidative stress accumulation, compared to MKN45 cells. In light of these findings, the higher HSF1 levels of 23132/87 cells could have a protective role in the survival of these primary gastric adenocarcinoma cells under stress challenges. Finally, it cannot be excluded that the increased proteasome activity of MKN45 is actually contributing to the reduced cytosolic levels of HSF1 in this cell line.

The other significant signature is represented by the higher nuclear levels of YY1 in the metastatic cell line, which we hypothesized could at least in part explain the higher levels of *UBC* expression in MKN45 cells compared to 23132/87 cells, but this hypothesis has been denied by the *YY1* silencing experiments (see below).

siRNA-mediated targeting of *YY1*, *HSF1* and *SP1* was effective at both mRNA and protein levels in 23132/87 and MKN45 cell lines. However, the knockdown of these transcription factors does not significantly affect the expression of the four Ub genes. The results of TF silencing herein obtained differ from those previously reported for HeLa cervical cancer cells, where YY1 regulates the basal *UBC* expression [15] and HSF1 drives the transcriptional induction of the *UBC* gene after proteotoxic and oxidative stress [16,21], while SP1 plays a role in the basal *UBC* expression [14,17], besides being involved in the glucocorticoid-mediated transcriptional induction of the *UBC* gene, in muscle and multiple myeloma cells [17,18]. This may be explained by a cancer cell-type specific role of YY1, HSF1 and SP1 in regulating basal Ub gene expression. The lack of effect on *UBC* and *UBB* gene transcription in *HSF1*-silenced gastric cancer cells may depend on the fact that HSF1 is a stress-activated TF, while for YY1 we evaluated different target genes in the si*YY1*-transfected cells and we found that they are differently affected by *YY1* knockdown. Among *BAX*, survivin and *c-MYC*, only the expression levels of the oncogene *c-MYC* were affected, showing a significant reduction after siRNA targeting *YY1*.

Several reports demonstrated the pro-survival role of *UBB* and *UBC* in cancer cells. Oh et al. [9] showed that knockdown of *UBB* in neuroblastoma, hepatocarcinoma, breast and prostate cancer cell lines led to an increase in p53 and cleaved PARP1 levels, induction of G2/M block, increase in the number of apoptotic cells and decrease in cancer cell proliferation rates. In addition, Tang et al. [10] demonstrated that the knockdown of *UBB* and *UBC* in A549 lung cancer cells led to a reduction in cancer cell viability, activation of the intrinsic pathway of apoptosis, decrease in NF-kB and p-Akt levels (after irradiation treatment) and reduction in the volume of A549-derived xenografts. Lastly, Kedves et al. [48] showed that concurrent loss of *UBB* and *UBC* expression in several gynecological tumor cell lines led to a consistent decrease in their viability and to an increased survival of tumor-injected mice. On these bases, we targeted *UBB* and *UBC* in 23132/87 and MKN45 GC cells and found that every treatment significantly decreased the mRNA levels of the target gene. At the protein level, we found that si*UBB*, si*UBC* and si*UBB*+si*UBC* transfections were able to cause a similar reduction in conjugated Ub levels in 23132/87 and MKN45 GC cells, with the most noticeable reduction detected in the double knockdown cells. As for the levels of free Ub, they were significantly reduced by the silencing of polyubiquitin genes, with the lowest Ub levels detected in the primary 23132/87 cells treated with si*UBB*+si*UBC*, while in the same cell line, the targeting of only one gene (*UBB* or *UBC*) had a lower but similar impact on the free Ub content. While in the metastatic cell line MKN45, the *UBB* gene appears to mainly contribute to Ub homeostasis, in fact the free Ub content in *UBB* silenced cells is similar to that found in the double knockdown cells. Moreover, the reduction in ubiquitinated H2A levels that can be appreciated after transfection of siRNAs targeting the polyubiquitin genes *UBB* and *UBC*, alone or in combination, further supports the efficacy of the silencing at the protein level. In fact, as reported by Dantuma et al. [49], a redistribution of Ub between the different pools occurs under stressful conditions and the deubiquitination of histones contributes to restoring the mono-Ub pool necessary to cope with cellular stress. We can speculate that gastric cancer cells 23132/87 and MKN45 attempted to counteract the siRNA-mediated Ub depletion in a similar way, i.e., by using the ubiquitinated histones as a “ready to use” reservoir to partially restore the free Ub pool. The use of this rescue mechanism seems more evident in the 23132/87 primary cells subjected to the double knockdown of *UBB* and *UBC* genes. Overall, H2Aub levels show the same layout as mono-Ub in both cell lines, consistent with them being a source of readily available free ubiquitin.

Of note, there was a significant upregulation of the *UBB* gene after si*UBC* treatment in 23132/87 cells, indicating that these cells try to compensate for the *UBC* downregulation through an increase in the transcription of the other poly-Ub gene, namely *UBB*.

Noteworthy, *UBB* silencing does not result in *UBC* induction to restore cellular Ub levels, suggesting that *UBC* knockdown is more detrimental for 23132/87 cell survival given its higher coding potential compared to *UBB*. In this light, targeting both poly-Ub genes could prevent primary GC cells from activating those survival mechanisms able to restore their Ub levels. Interestingly, while MKN45 viability was not affected by *UBB* and *UBC* knockdown, 23132/87 suffered a cell viability reduction after treatment with a combination of si*UBB* and si*UBC*, which caused an increase in the tumor suppressor protein Fas and activation of the apoptotic process. In agreement with the lack of cell viability reduction in the metastatic MKN45 cells, neither Fas nor caspase 3 levels were modified by the si*UBB*+si*UBC* treatment. The role of extrinsic apoptosis in the reduction in cell viability of GC cells has been investigated by Hsu et al. [39]. The authors showed that the activation of the apoptotic process reduced the proliferation rate of AGS and SNU-16 GC cells, particularly after Fas ligand (FasL) treatment and activation of Fas [39]. The interaction of FasL with the transmembrane protein Fas indeed leads to the activation of caspase 8 and caspase 3, inducing the extrinsic apoptotic pathway [28,50]. Apoptosis is a tightly controlled process, characterized by three pathways, namely, the mitochondrial [28], endoplasmic reticulum [51] and death receptor signaling pathways [28]. Interestingly, Zhang et al. have demonstrated, in several cancer cell lines, that the oncoprotein Cysteine-rich intestinal protein 1 (CRIP1) can interact with Fas, enhancing its ubiquitination and degradation and leading to inhibition of caspases 8 and 3, thus underlining the tumor suppressor role of this transmembrane protein [52].

Interestingly, the role of the oncoprotein β-catenin in overproliferation, migration and invasion of several tumors, including gastric adenocarcinoma, has been reported [40]. We found that the siRNA-mediated knockdown of both *UBB* and *UBC* mRNAs decreases the β-catenin levels in 23132/87 cells, but not in MKN45 cells. The reduction in β-catenin levels correlates with the cell viability decrease after si*UBB*+si*UBC* treatment detected only in the primary 23132/87 gastric cancer cells. These data complement the evidence by Zhang et al. [40] and further consolidate the role of β-catenin as an important factor in the “survivability fitness” of these cell lines, in particular for MKN45 cells, which are able to preserve steady levels of this metastatic marker.

Results herein obtained show that the combined silencing of *UBB* and *UBC* genes triggers the molecular pathways of extrinsic apoptosis only in 23132/87 cells, suggesting that Ub downmodulation may counteract the pro-survival role of poly-Ub genes in primary but not in metastatic gastric adenocarcinoma. This different sensitivity to reduction in Ub levels of the primary and metastatic GC cell lines cannot be explained by a different intracellular Ub content/distribution between pools but rather by a different dependence on Ub levels for survival and proliferation.

Up- and downregulation of Ub content has been demonstrated to affect the levels/activity of many regulatory proteins in a cell- and context-specific manner [53,54]. Furthermore, little is known about the trans-acting factors controlling Ub gene transcription in cancer cells.

Our data allow us to exclude the involvement of YY1, HSF1 and Sp1 in driving Ub expression in 23132/87 and MKN45 GC cell lines. Thus, further studies on the molecular players involved in the control of Ub gene expression as well as on signaling cascades affected by altered Ub levels will be of value for the characterization of the molecular patterns associated with GC development.

Meanwhile, the role of Ub in the regulation of intracellular levels of various tumor suppressor proteins should be addressed in GC, as already reported for BAX [55] and p53 [56] in other cancers. In a very recent paper, Peng et al. [55] showed that mutation of the two ubiquitin-binding sites in the pro-apoptotic protein BAX increased its half-life and its ability to activate the intrinsic pathway of apoptosis in HCT116 tumor cells. Zhou et al. [56] demonstrated that the oncoprotein MNAT1 binds to p53 leading to its ubiquitin-mediated degradation through Mouse double minute 2 homolog (MDM2), decreasing apoptosis and increasing cell growth in human colorectal cancers. On these bases, it would be important to investigate if decreasing the Ub levels in GC through the targeting of the poly-Ub coding genes affects the levels of such apoptotic markers. Our preliminary evidence shows that combined targeting of poly-Ub genes *UBB* and *UBC* significantly affects the cellular Ub protein content in the two GC cell lines, being more detrimental for the viability of primary gastric adenocarcinoma 23132/87 cells. Further in vitro experiments (i.e., using other GC cell lines such as AGS and SNU-16 [39]) and in vivo studies are needed to warrant *UBB* and *UBC* as potential therapeutic targets and Ub protein levels as a new biomarker in gastric adenocarcinoma.

## 4. Materials and Methods

### 4.1. Cell Cultures and Chemicals

23132/87 and MKN45 GC cell lines were purchased from DMSZ (German Collection of Microorganisms and Cell Cultures GmbH; Braunschweig, Germany). 23132/87 cells have been identified as primary gastric adenocarcinoma of the intestinal type [57], while MKN45 have been identified as a metastatic GC cell line displaying the characteristics of both diffuse and intestinal types [58]. The web reference “https://cansarblack.icr.ac.uk/cell-line/MKN-45/copy-number” [59] reports average copy numbers of 1.132, 1.091 and 1.104 for *HSF1*, *YY1* and *SP1* in MKN-45, respectively, and 1.100, 1.094, 1.061 and 1.127 for *UBC*, *UBB*, *UBA52* and *RPS27A*, respectively. MKN45 cells are also reported by the DSMZ site as a hypertryploid cell line, with 8% of polyploidy; the percentage seems to roughly correspond with the average copy numbers reported above. 23132/87 cells were grown at 5% CO_2_ at 37 °C in Roswell Park Memorial Institute (RPMI) 1640 medium supplemented with 20% heat-inactivated fetal bovine serum (Sigma-Aldrich, St. Louis, MO, USA), 2 mM glutamine and 1x antibiotics (100 µg/mL streptomycin and 100 U/mL penicillin), both from Sigma-Aldrich. MKN45 cells were grown at 5% CO_2_ at 37 °C in the same medium, but 20% heat-inactivated fetal bovine serum (10270-106) from Gibco (ThermoFisher Scientific, Waltham, MA, USA) was used instead. All cell lines were sub-cultivated at a 1:3 ratio, 3 times per week. The 23132/87 and MKN45 GC cell lines were proved to be mycoplasma-free (every 6 months) using the EZ-PCR Mycoplasma Test Kit (BI, Biological Industries). The most recent Mycoplasma Test assay was performed on January 17th, 2020 (Figure S1D). All chemicals were purchased from Sigma-Aldrich, unless otherwise specified.

### 4.2. Small Interfering RNA Transfection in 23132/87 and MKN45 Cells

Small interfering RNA (siRNA)-mediated gene silencing in 23132/87 and MKN45 cells was achieved by transfecting siRNA duplexes with RNAiMAX (Invitrogen, Thermo Fisher Scientific), according to the standard transfection protocol. Briefly, 3 × 10^5^ and 6 × 10^5^ cells for 23132/87 and MKN45, respectively, were seeded in 6-well plates, while 5 × 10^3^ cells were seeded in 96-well plates for both cell lines. After 24 h, siRNAs targeting *UBB*, *UBC* (or their combination at 10 nM si*UBB* and 10 nM si*UBC*), *YY1*, *HSF1* or *SP1* mRNA and the *GFP*-targeting control siRNA were transfected at a final concentration of 20 nM in the presence of 9 µL and 1.5 µL of RNAiMAX transfection reagent for 6-well plates and 96-well plates, respectively. The samples were collected after 48 h. *HSF1* siRNA was purchased from Qiagen (Hilden, Germany); *SP1*, *UBB* and *UBC* siRNAs were purchased from Sigma-Aldrich, while the *YY1* siRNA and *GFP* control siRNA were from Biomers (Ulm, Germany). The oligonucleotide targeting sequences are reported below:

Hs_*YY1*: ATGCCTCTCCTTTGTATATTA
Hs_*HSF1*: CAGGTTGTTCATAGTCAGAAT
Hs_*SP1*: TTGGGTAAGTGTGTTGTTTAA
Hs_*UBB*: CCAAGATCCAAGATAAAGA
Hs_*UBC*: GATCAGCAGAGGTTGATCT
*GFP*: CGGCAAGCTGACCCTGAAGTTCAT

### 4.3. Real-Time Quantitative Polymerase Chain Reaction (RT-qPCR)

For gene-specific expression analysis, total RNA was isolated using the RNeasy Plus Mini kit (Qiagen, Hilden, Germany). A quantity of 0.5 µg of total RNA was reverse-transcribed using Primescript RT Master Mix (Perfect Real Time; Takara Bio Europe SAS, Saint-Germain-en-Laye, France) with oligo-dT and random hexamer primers, following the manufacturer’s instructions. qPCR detection and expression analysis of genes was performed with Synergy Brands (SYBR) green quantitative real-time PCR, using the Hot-Rescue Real Time PCR Kit (Diatheva s.r.l., Cartoceto PU, Italy), according to the manufacturer’s instructions. Briefly, the reaction was set up in a 25 µL final volume, using 5 ng cDNA as the template and 200 nM of each specific primer. For RT-qPCR amplifications, 40 PCR cycles were run with the following thermal profile: 15 s at 95 °C melting temperature, 15 s at 60 °C annealing and 1 min at 72 °C extension temperature per cycle; before cycling, 10 min at 95 °C were allowed for Hot-Rescue Taq DNA polymerase activation. Fluorescence intensity of each amplified sample was measured with an ABI PRISM 7700 Sequence detection system (Applied Biosystems, Foster City, CA, USA). All measurements were performed at least in triplicate and reported as the average values ± standard deviation of the mean (mean ± SD). Target gene values were normalized with *B2M* mRNA measurements, and expression data were calculated according to the 2^−ΔΔCt^ method [60]. Primers were designed using Primer 3 Plus, and their sequences are reported in Table 1.

### 4.4. Cell Extracts

To obtain whole protein extracts, cells were scraped from plates with a buffer containing 50 mM Tris/HCl pH 7.8, 0.25 M sucrose, 2% (*w*/*v*) sodium dodecyl sulfate (SDS), 10 mM N-ethylmaleimide (NEM), 1 mM NaF and 1 mM Na_3_VO_4_, supplemented with a cocktail of protease inhibitors (Roche Diagnostics, Mannheim, Germany). Lysates were boiled, sonicated twice at 100 Watts for 10 sec and cleared by centrifugation at 12,000× *g* for 10 min, then the supernatant was recovered. To obtain cytosolic and nuclear extracts, after washing with phosphate buffered saline (PBS), cells were scraped from the dishes with cold buffer A (10 mM HEPES/KOH pH 7.9, 1.5 mM MgCl_2_, 1 mM NEM, 1 mM ethylenediaminetetraacetic acid (EDTA), 10 mM KCl, 0.1% (*v*/*v*) Nonidet-P40, 0.5 mM dithiotreitol (DTT), 1 mM NaF and 1 mM Na_3_VO_4_, supplemented with protease inhibitors (Roche Diagnostics). The samples were then incubated on ice for 10 min before centrifugation at 12,000 × *g* for 10 min at 4 °C. Supernatants (containing cytosolic proteins) were recovered, while the nuclear pellets were lysed in the same buffer used to obtain whole protein extracts, then boiled, sonicated and cleared by centrifugation at 12,000× *g* for 10 min to obtain nuclear fractions. The protein content in whole cell extracts and nuclear fractions was determined by the method of Lowry, while the Bradford assay (Bio-Rad, Hercules, CA, USA) was used for the cytosolic fractions.

### 4.5. Western Blot Analysis

Proteins were resolved by SDS polyacrylamide gel electrophoresis (SDS-PAGE) and electroblotted onto a nitrocellulose membrane (0.2 μm pore size) (Bio-Rad). The blots were probed with the following primary antibodies: anti-HSF1 (#4356, polyclonal), anti-Fas (#4233, monoclonal: C18C12), anti-Lamin A/C (#4777, monoclonal: 4C11; for nuclear extracts), anti-Caspase 3 (#9668, monoclonal: 3G2) and anti-H2AUb (Lys 119) (#8240, monoclonal: D27C4) from Cell Signaling Technology (Danvers, MA, USA); anti-YY1 (#sc-7341, monoclonal: H-10), anti-SP1 (#sc-420, monoclonal: 1C6), anti-Ub (rabbit polyclonal, kindly provided by Prof. A. L. Haas, Louisiana State University, Health Sciences Center, New Orleans), anti-β-Actin (#sc-47778, monoclonal: C4; for whole extracts) and anti-β-catenin (#sc-7963, monoclonal: E-5) from Santa Cruz Biotechnology (Dallas, TX, USA); anti-GAPDH (#A300-641A-T, polyclonal; for cytosolic extracts) from Bethyl Laboratories (Montgomery, TX, USA). Immunoreactive bands were detected by horseradish peroxidase (HRP)-conjugated secondary antibodies (Bio-Rad). Peroxidase activity was detected with the enhanced chemiluminescence detection method (WesternBright ECL, Advasta, Menlo Park, CA, USA) using the ChemiDoc MP Imaging System (Bio-Rad). Quantification of the protein bands was performed using Image Lab analysis software version 5.2.1 (Bio-Rad).

### 4.6. Cell Viability Assay

The effect of siRNA transfections (performed in 96-well plates as detailed above) on cell viability was evaluated by using the CellTiter 96 AQueous One Solution Cell Proliferation Assay (Promega s.r.l., Milan, Italy). This assay is based on the reduction of the MTS reagent [3-(4,5-dimethylthia-zol-2yl)-5-(3-carboxymethoxyphenyl)-2-(4-sulfophenyl)2*H*-tetrazolium, inner salt] into a colored formazan product that is soluble in culture medium. This conversion is accomplished by NADPH or NADH produced by dehydrogenase enzymes in metabolically active cells. The quantity of formazan product, measured by absorbance at 490 nm, is directly proportional to the number of living cells in the culture. The results were expressed as a percentage of residual cell viability compared to control cells treated with 20 nM si*GFP* (set at 100% cell viability) for both 23132/87 and MKN45 cell lines.

### 4.7. Proteasome Activity Assay

The chymotrypsin-like activity of the 20S proteasome was measured in 23132/87 and MKN45 GC cell lysates using the fluorogenic substrate *N*-succinyl-Leu-Leu-Val-Tyr-7-amido-4- methylcoumarin (sLLVY-NH-Mec, from Sigma-Aldrich, St. Louis, MO, USA) as previously described [21]. Briefly, untreated 23132/87 and MKN45 cells were homogenized on ice in a buffer consisting of 50 mM HEPES/KOH pH 7.8, 1 mM DTT and 0.25 M sucrose. Then, 25, 50 and 75 µg of cleared extracts were incubated at 37 °C in 100 mM HEPES/KOH buffer, pH 7.8, 5 mM MgCl_2_ and 10 mM KCl and the reaction was initiated by addition of 0.2 mM fluorogenic substrate. The breakdown of the peptide was monitored using a fluorescence microplate reader (FLUOstar OPTIMA, BMG Labtech GmbH, Offenburg, Germany) with an excitation wavelength of 355 nm and an emission wavelength of 460 nm. Proteasome activity in each sample, expressed as fluorimetric units/min/mg, was calculated by submitting data to linear regression analysis (R^2^ > 0.99).

### 4.8. Ubiquitin Carboxyl-Terminal Hydrolase 2 (Usp2) Digestion and Mono-Ubiquitin Quantification

23132/87 and MKN45 cells were washed with ice cold PBS and lysed in a buffer consisting of 20 mM Hepes/KOH pH 7.9, 25% glycerol, 0.42 M NaCl, 1.5 mM MgCl_2_ and 1% Nonidet P40, supplemented with a cocktail of protease inhibitors. After 20 min incubation on ice, cell extracts were cleared by centrifugation and protein content was determined by the Bradford assay. Twenty micrograms of extract were incubated at 37 °C for 90 min in a water bath with 0.5 μg of recombinant Usp2 protein or without Usp2 addition (for the undigested control), in a final volume of 40 μL. The digestions were stopped by adding an equal volume of SDS-PAGE sample buffer and boiling. The effective deconjugation of Ub after Usp2 treatment was always verified by running undigested and digested extracts in parallel. To quantify total Ub protein levels, different amounts of Usp2-treated extracts were run on the same gel, in parallel with different amounts of purified ubiquitin (Sigma-Aldrich) used as the reference standard, and submitted to western immunoblotting analysis with an antibody against Ub [53]. The adjusted volume intensity of the ubiquitin immunoreactive bands, in both Ub standard and sample cell lines, was determined using Image Lab analysis software version 5.2.1. Calibration curves were generated, for each immunoblot, by plotting band intensities (adjusted volumes) against Ub standard concentrations. A regression line equation was then generated and used to calculate the Ub concentration in the cell protein samples. The coefficient of determination (R^2^) for ubiquitin standard curves was always in the range 0.98–0.99.

### 4.9. Statistical Analysis

The data were expressed as mean ± SD from at least three independent experiments. Student’s *t*-test performed with GraphPad Prism Software version 3.06 (La Jolla, CA, USA) was used for statistical analysis of the data; differences between groups were considered statistically significant when *p* < 0.05. The actual *p*-values have been reported in the text.

## Figures and Tables

**Table 1:** Oligonucleotides used for quantitative real-time PCR.

Forward Primer	Sequence (5′ to 3′)	Reverse Primer	Sequence (5′ to 3′)
UBC-f	GTGTCTAAGTTTCCCCTTTTAAGG	UBC-r	TTGGGAATGCAACAACTTTATTG
UBB-f	CTTTGTTGGGTGAGCTTGTTTGT	UBB-r	GACCTGTTAGCGGATACCAGGAT
UBA52-f	CTGCGAGGTGGCATTATTGAG	UBA52-r	GTTGACAGCACGAGGGTGAAG
RPS27A-f	TCGTGGTGGTGCTAAGAAAAGG	RPS27A-r	TTCAGGACAGCCAGCTTAACCT
B2M-f	GCCTGCCGTGTGAACCAT	B2M-r	CATCTTCAAACCTCCATGATGCT
HSF1-f	CTGACGGACGTGCAGCTGAT	HSF1-r	CCCGCCACAGAGCCTCAT
YY1-f	GAAGCCCTTTCAGTGCACGTT	YY1-r	ACATAGGGCCTGTCTCCGGTAT
SP1-f	GCCTCTCAACTGCCCTAAGTCCT	SP1-r	ACCTGCCCTTGTCCACAATGTT
C-MYC-f	CTGAAGAGGACTTGTTGCGGAAAC	C-MYC-r	TCTCAAGACTCAGCCAAGGTTGTG

## Supplementary Materials

Supplementary materials can be found at https://www.mdpi.com/1422-0067/21/15/5435/s1. Figure S1. Characterization of 23132/87 and MKN45 gastric adenocarcinoma cells. Figure S2. Quantification of total ubiquitin content in 23132/87 and MKN45 gastric cancer cells. Table S1. Fold changes of *UBC*, *UBB*, *UBA52* and *RPS27A* expression levels in 23132/87 and MKN45 gastric cancer cells, after normalization with *GAPDH* mRNA levels. Table S2. Fold changes of *C-MYC* expression levels in 23132/87 and MKN45 gastric cancer cells, after *YY1* silencing. Table S3. Quantification of free Ub content in 23132/87 and MKN45 gastric cancer cells, after *UBB*, *UBC* and *UBB*+*UBC* silencing.

## Abbreviations

B2M	Beta-2-Microglobulin
BAX	Bcl-2-like protein 4
C-MYC	Avian myelocitomatosis virus oncogene cellular homolog
CRIP1	Cysteine-rich intestinal protein 1
DTT	Dithiotreitol
DUB	Deubiquitinase
EDTA	Ethylenediaminetetraacetic acid
EMT	Epithelial to mesenchymal transition
FasL	Fas ligand
GAPDH	Glyceraldehyde 3-phosphate dehydrogenase
GC	Gastric cancer
GFP	Green fluorescent protein
HRP	Horseradish peroxidase
HSF1	Heat shock factor 1
KLF4	Kruppel-like factor 4
LUCAT1	Lung cancer-associated transcript 1
MDM2	Mouse double minute 2 homolog
MTS	3-(4,5-dimethylthia-zol-2yl)-5-(3-carboxymethoxyphenyl)-2-(4-sulfophenyl)2*H*-tetrazolium
NEM	*N*-ethylmaleimide
PARP1	Poly(ADP-ribose) polymerase 1
PBS	Phosphate buffered saline
RPS27A	Ribosomal protein s27a
RT-qPCR	Real-time quantitative polymerase chain reaction
SDS	Sodium dodecyl sulfate
SDS-PAGE	SDS polyacrylamide gel electrophoresis
siRNA	Small interfering RNA
SP1	Specificity protein 1
SYBR	Synergy Brands
TF	Transcription factor
Ub	Ubiquitin
UBB	Ubiquitin B
UBA52	Ubiquitin a-52 residue ribosomal protein fusion product 1
UBC	Ubiquitin C
Usp2	Ubiquitin carboxyl-terminal hydrolase 2
YY1	Yin yang 1
