# Supplementary material for: The Ubiquitin Gene Expression Pattern and Sensitivity to UBB and UBC Knockdown Differentiate Primary 23132/87 and Metastatic MKN45 Gastric Cancer Cells
Source: Int J Mol Sci. 2020 Jul 30;21(15):5435. doi: 10.3390/ijms21155435 (PMC7432825; doi:10.3390/ijms21155435)
Supplement: Supplementary file 1 [file ijms-21-05435-s001.pdf]

SUPPLEMENTAL DATA

The ubiquitin gene expression pattern and sensitivity to *UBB* and *UBC* knockdown differentiate primary 23132/87 and metastatic MKN45 gastric cancer cells

Emanuele-Salvatore Scarpa[1,*], Filippo Tasini[1], Rita Crinelli[1], Chiara Ceccarini[1], Mauro Magnani[1#], Marzia Bianchi[1#]
# Both authors contributed equally to this Manuscript

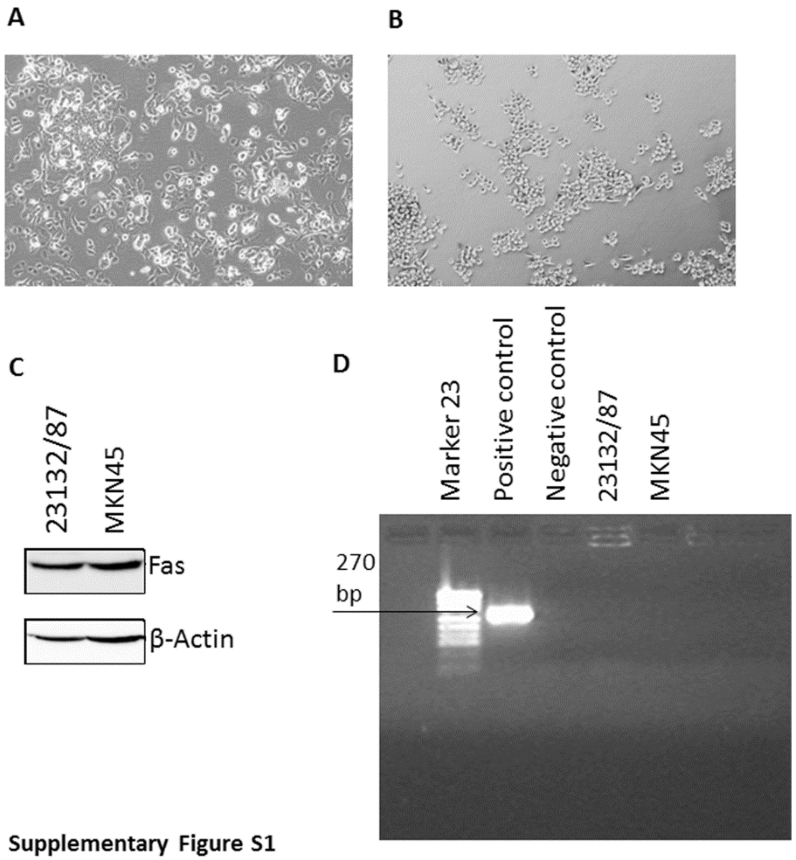

Figure S1. Characterization of 23132/87 and MKN45 gastric adenocarcinoma cells. Representative images of 23132/87 (A) and MKN45 (B) were acquired using Olympus IX51 microscope at 20x magnification. (C) Representative western immunoblots (n=3) performed with anti-Fas antibody using 15 µg of total lysates extracted from 23132/87 and MKN45 cell lines; blots were reprobed with anti ß-Actin antibody, used as loading control. (D) Mycoplasma detection assay performed in 23132/87 and MKN45 GC cells; Marker 23 stands for pUC19 DNA/MspI (HpaII) (Thermo Fisher Scientific, Waltham, MA, USA).

Materials and Methods

Cell extracts

To obtain whole protein extracts, cells were scraped from plates with a buffer containing 50 mM Tris/HCl pH 7.8, 0.25 M sucrose, 2% (w/v) SDS, 10 mM N-ethylmaleimide (NEM), 1 mM NaF, 1 mM Na_3VO_4, supplemented with a cocktail of protease inhibitors (Roche Diagnostics, Mannheim, Germany). Lysates were boiled, sonicated twice at 100 Watts for 10 sec and cleared by centrifugation at 12000 x *g* for 10 min, then the supernatant was recovered.

Western blot analysis

Proteins were resolved by SDS polyacrylamide gel electrophoresis (SDS PAGE) and electroblotted onto a nitrocellulose membrane (0.2 µm pore size) (Bio-Rad). The blots were probed with the following primary antibodies: anti-Fas (#4233, monoclonal: C18C12) from Cell Signaling Technology (Danvers, MA, USA); anti-β-Actin (#sc-47778, monoclonal: C4; for whole extracts) from Santa Cruz Biotechnology (Dallas, TX, USA). Immunoreactive bands were detected by horseradish peroxidase (HRP)-conjugated secondary antibodies (Bio-Rad). Peroxidase activity was detected with the enhanced chemiluminescence detection method (WesternBright ECL, Advasta, Menlo Park, CA, USA) using ChemiDoc MP Imaging System (Bio-Rad).

Mycoplasma detection assay

Both cultured cell lines were tested for Mycoplasma contamination at least once every six months and proven Mycoplasma-free using EZ-PCR Mycoplasma Test Kit (Biological Industries, Beit Haemek, Israel), according to the manufacturer instructions.

A

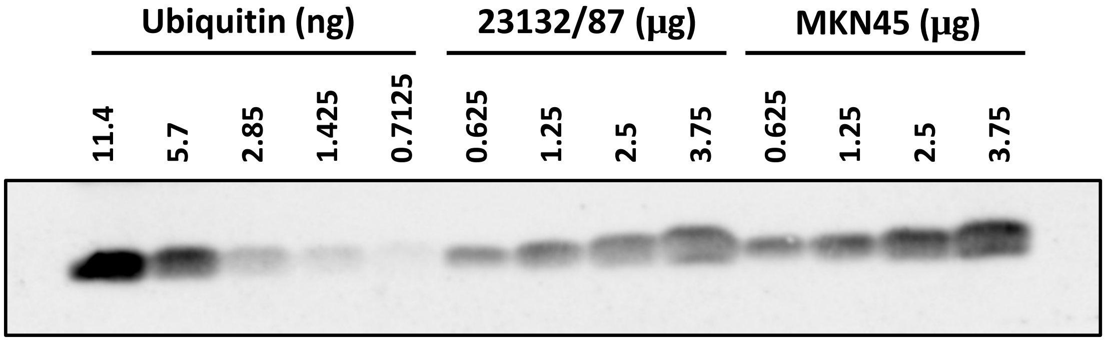

B

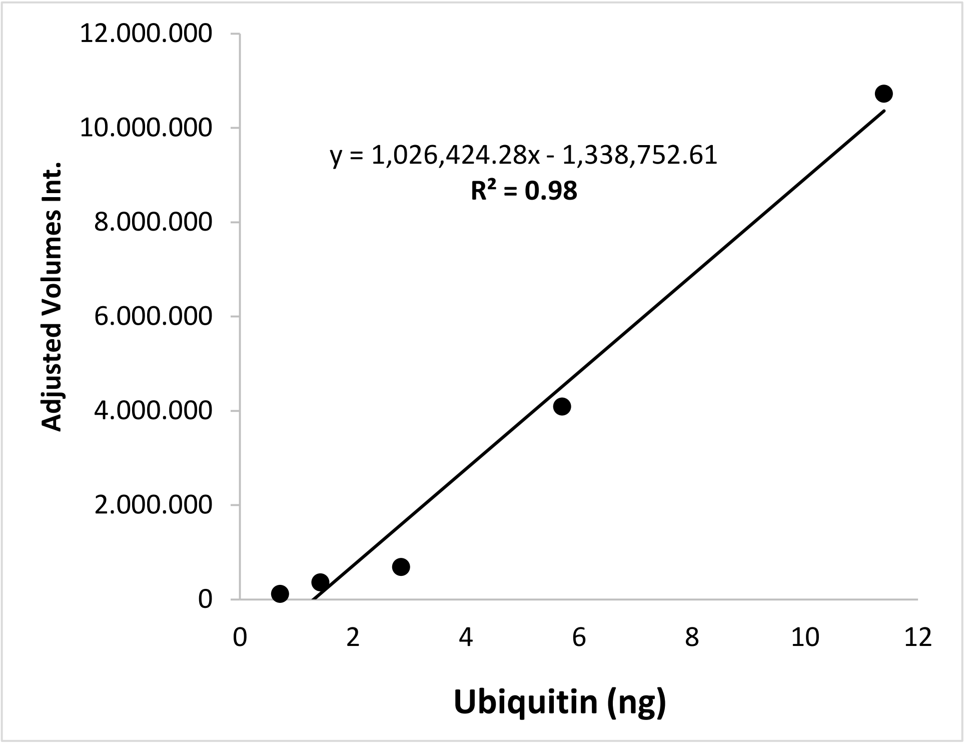

Ubiquitin (ng)	Adj. Vol. (Int)
11.4000	10,722,923
5.7000	4,090,443
2.8500	689,118
1.4250	359,600
0.7125	115,299

Figure S2. Quantification of total ubiquitin content in 23132/87 and MKN45 gastric cancer cells. (A) A representative immunoblot used for the absolute quantification of total Ub. Whole protein extracts were obtained as described in the Materials and Methods section 4.8. Different amounts of Usp2-treated protein samples were loaded for both 23132/87 and MKN45 cells; Ub standards were provided in the same immunoblot, as indicated. (B) Standard curve derived from the Ub standards of the immunoblot by plotting band intensities (adjusted volumes) against Ub concentrations (the values used are shown in the table on the right). The equation generated by linear regression and the coefficient of determination (R^2) are shown.

Materials and Methods

Ubiquitin carboxyl-terminal hydrolase 2 (Usp2) digestion and mono-ubiquitin quantification

23132/87 and MKN45 cells were washed with ice cold PBS and lysed in a buffer consisting of 20 mM Hepes/KOH pH 7.9, 25% glycerol, 0.42 M NaCl, 1.5 mM $MgCl_2$, 1% Nonidet P40, supplemented with a cocktail of protease inhibitors. After 20 min incubation on ice, cell extracts were cleared by centrifugation and protein content was determined by the Bradford assay. Twenty µg of extract were incubated at 37°C for 90 min in a water bath with 0.5 µg of recombinant Usp2 protein or without Usp2 addition (for the undigested control), in a final volume of 40 µl. The digestions were stopped by adding an equal volume of SDS-PAGE sample buffer and boiling. The effective deconjugation of Ub after Usp2 treatment has been always verified by running undigested and digested extracts in parallel. To quantify total Ub protein levels, different amounts of Usp2-treated extracts were run on the same gel, along with different amounts of purified ubiquitin (SIGMA Aldrich) used as reference standard, and submitted to western immunoblotting analysis with an antibody against Ub. The adjusted volume intensity of the ubiquitin immunoreactive bands, in both Ub standard and cell sample lanes, was determined using Image Lab analysis software version 5.2.1. Calibration curves were generated, for each immunoblot, by plotting band intensities (adjusted volumes) against Ub standard concentrations. A linear regression equation was then generated and used to calculate the Ub concentration in the cell protein samples. Only Ub standard amounts fitting in the linear range were plotted. The coefficient of determination (R^2) for the ubiquitin standard curves was always between 0.98-0.99.

Western blot analysis

Proteins were resolved by SDS polyacrylamide gel electrophoresis (SDS PAGE) and electroblotted onto a nitrocellulose membrane (0.2 µm pore size) (Bio-Rad). The blots were probed with the following primary antibody: anti-Ub (rabbit polyclonal, kindly provided by Prof. A. L. Haas, Louisiana State University, Health Sciences Center, New Orleans) . Immunoreactive bands were detected by horseradish peroxidase (HRP)-conjugated secondary antibodies (Bio-Rad). Peroxidase activity was detected with the enhanced chemiluminescence detection method (WesternBright ECL, Advasta, Menlo Park, CA, USA) using ChemiDoc MP Imaging System (Bio-Rad).

Supplementary Information for

The ubiquitin gene expression pattern and sensitivity to *UBB* and *UBC* knockdown differentiate primary 23132/87 and metastatic MKN45 gastric cancer cells

Authors: Emanuele-Salvatore Scarpa[1*], Filippo Tasini[1], Rita Crinelli[1], Chiara Ceccarini[1], Mauro Magnani[1#], Marzia Bianchi[1#]

# Both authors contributed equally to this Manuscript

[1]Department of Biomolecular Sciences

University of Urbino *Carlo Bo*, 61029 - Urbino (PU), Italy

* Correspondence: emanuele.scarpa@uniurb.it; Tel.: +39 0722 305252

SUPPLEMENTARY FIGURE LEGENDS

Supplementary Figure S3. Full-length immunoblots for results shown in Figure 1 and Figure 2.
Lanes highlighted by a star represent samples unrelated to this study.

Supplementary Figure S4. Full-length immunoblots for results shown in Figure 2 and Figure 3.
Lanes highlighted by a star represent samples unrelated to this study.

Supplementary Figure S5. Full-length immunoblots for results shown in Figure 3.
Lanes highlighted by a star represent samples unrelated to this study.

Supplementary Figure S6. Full-length immunoblots for results shown in Figure 4 .

Supplementary Figure S7. Full-length immunoblots for results shown in Figure 5.
Lanes highlighted by a star represent samples unrelated to this study.

Supplementary Figure S8. Full-length immunoblots for results shown in Figure 5 and Supplementary Figure S1.
Lanes highlighted by a star represent samples unrelated to this study.

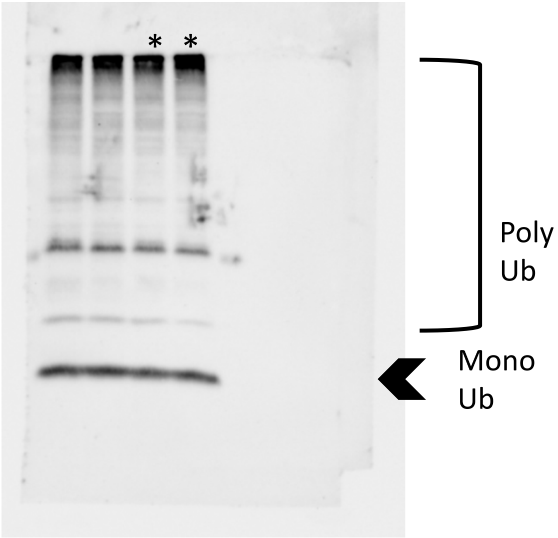

Fig 1C

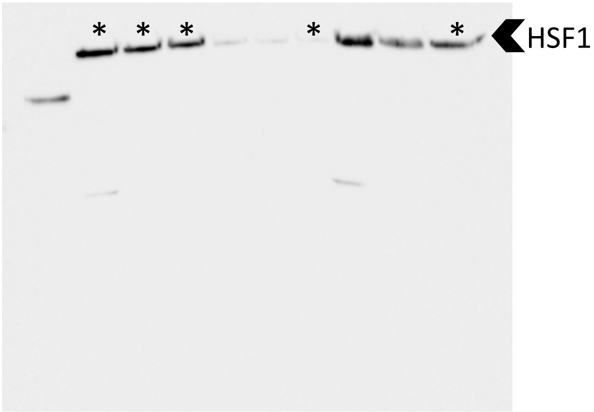

Fig 2A, 2C, 2E

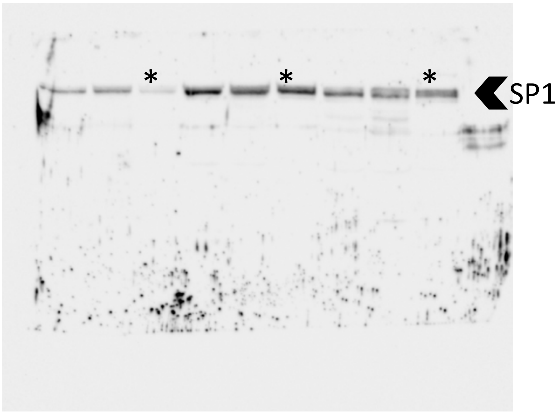

Fig 2A, 2C, 2E

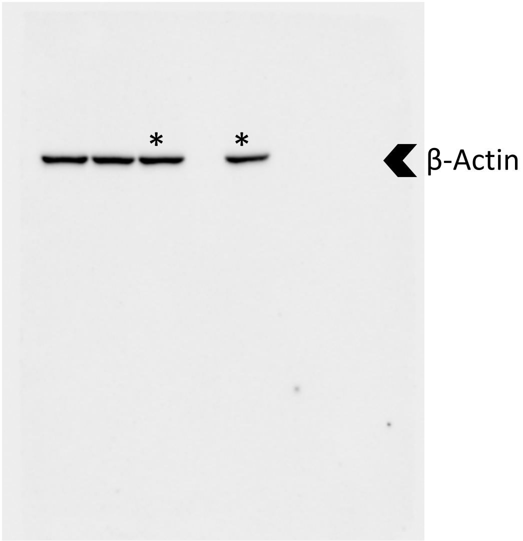

Fig 1C

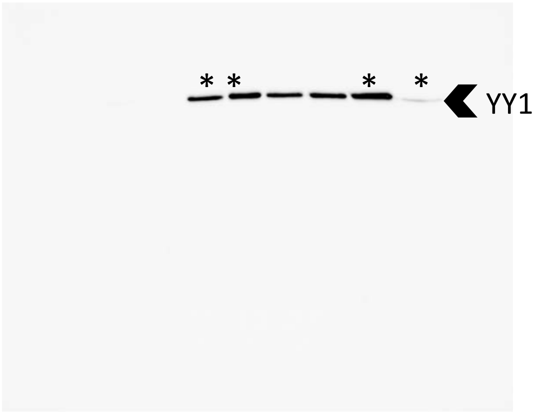

Fig 2A

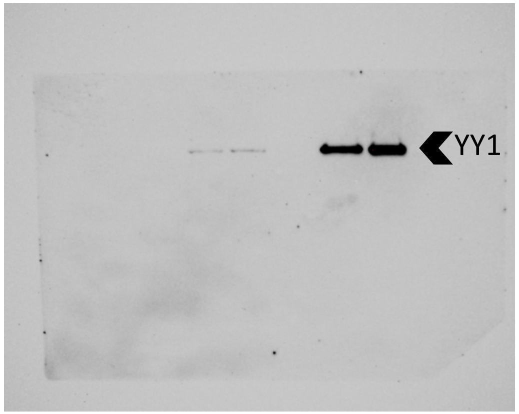

Fig 2C, 2E

Supplementary Figure S3

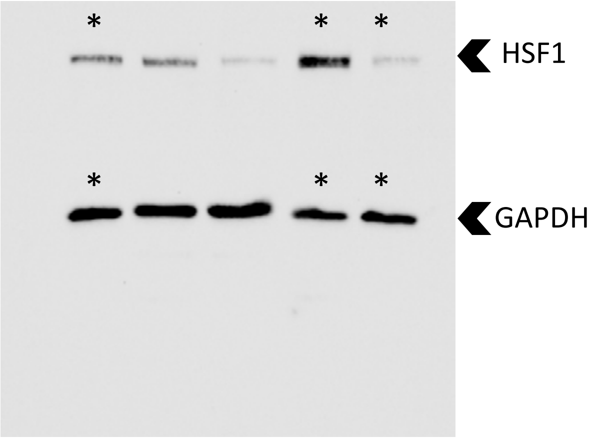

Fig 2C

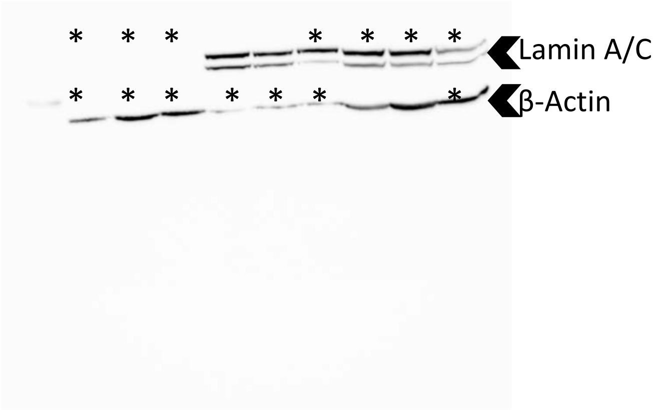

Fig 2A, 2E

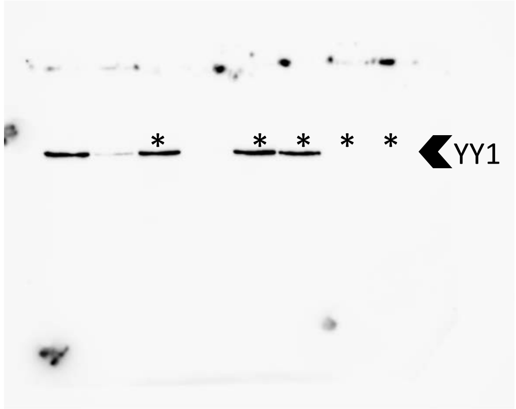

Fig 3B

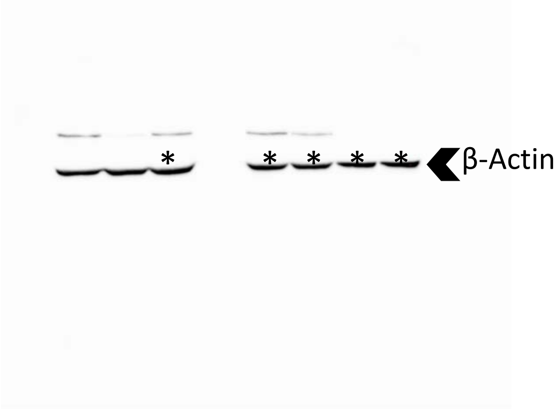

Fig 3B

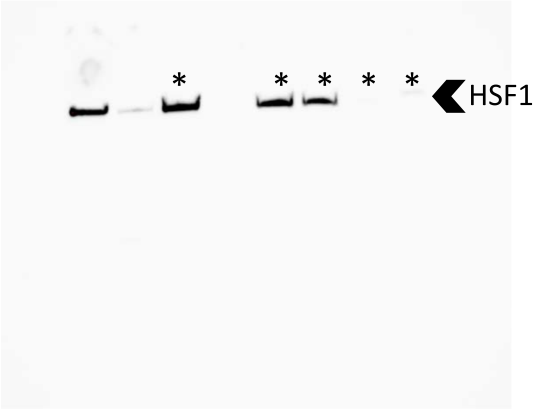

Fig 3C

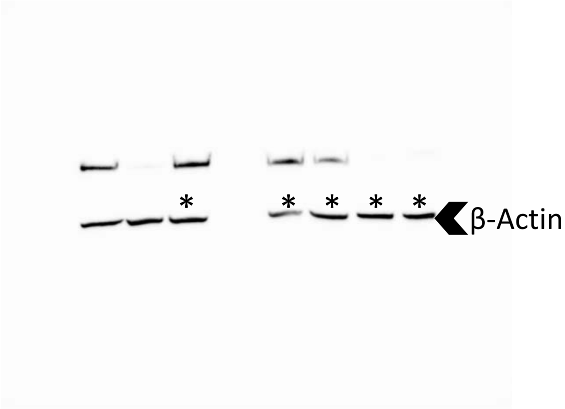

Fig 3C

Supplementary Figure S4

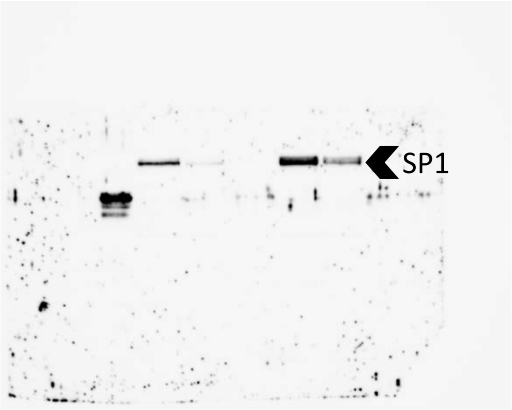

Fig 3D, 3H

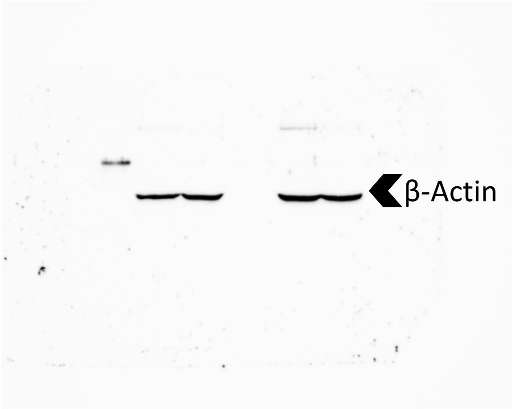

Fig 3D, 3H

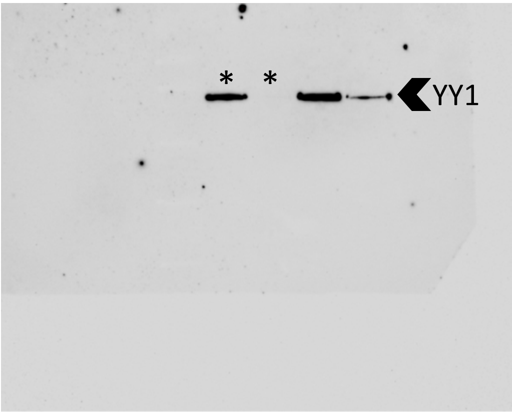

Fig 3F

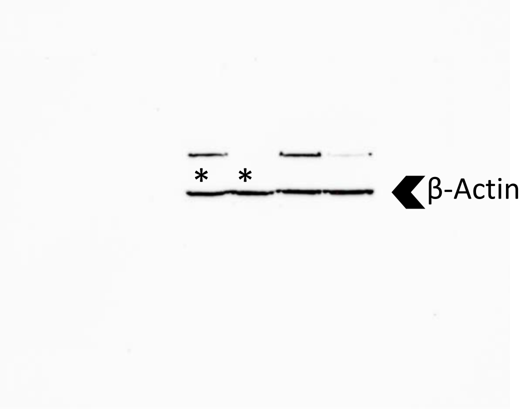

Fig 3F

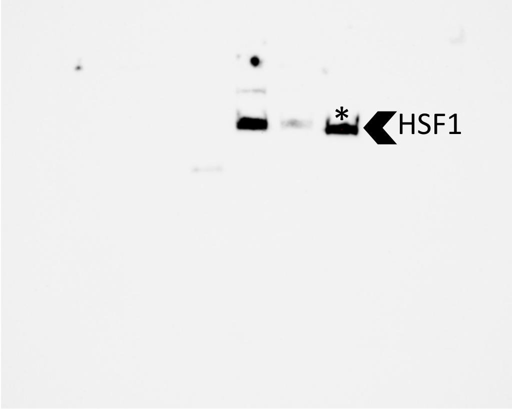

Fig 3G

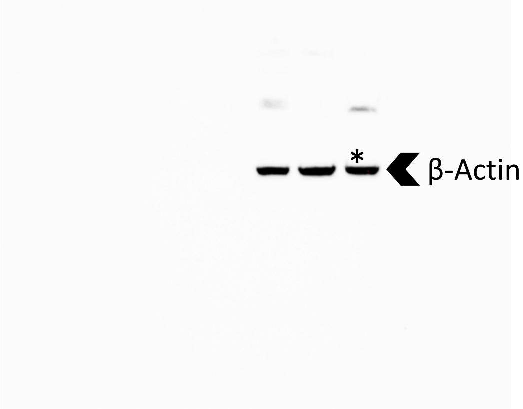

Fig 3G

Supplementary Figure S5

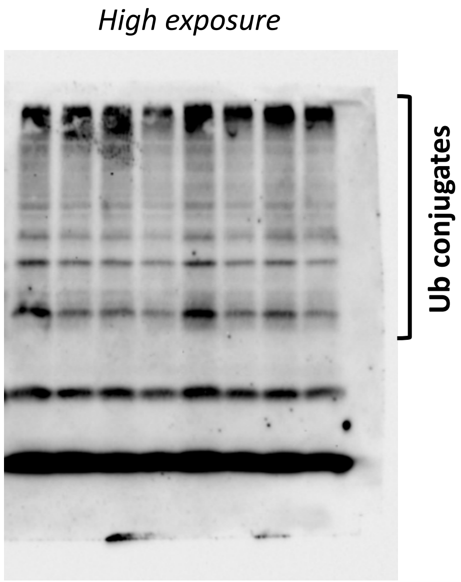

Fig 4C

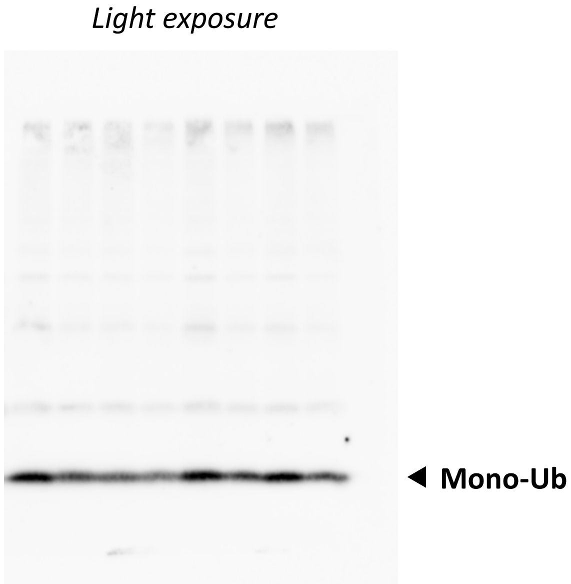

Fig 4C

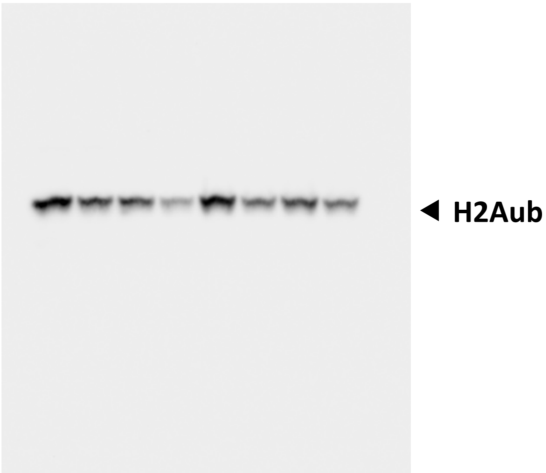

Fig 4C

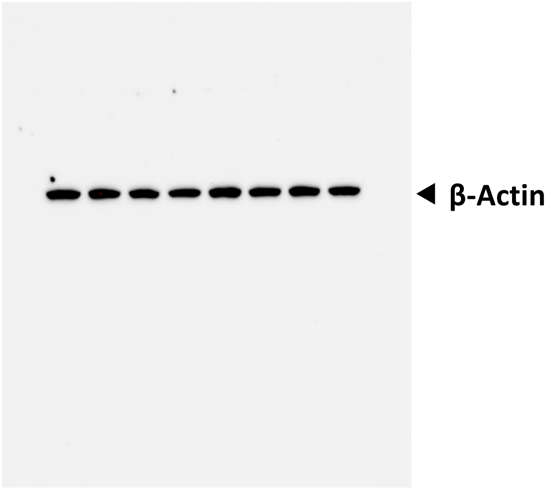

Fig 4C

Supplementary Figure S6

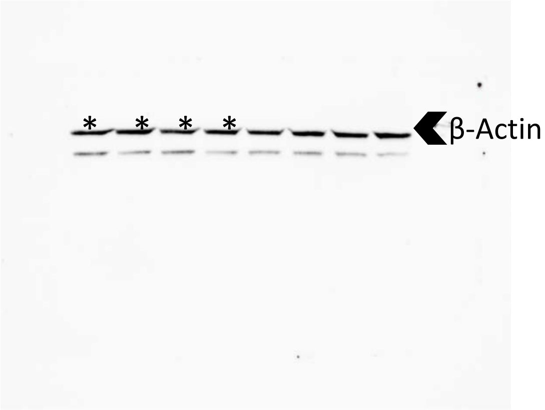

Fig 5C

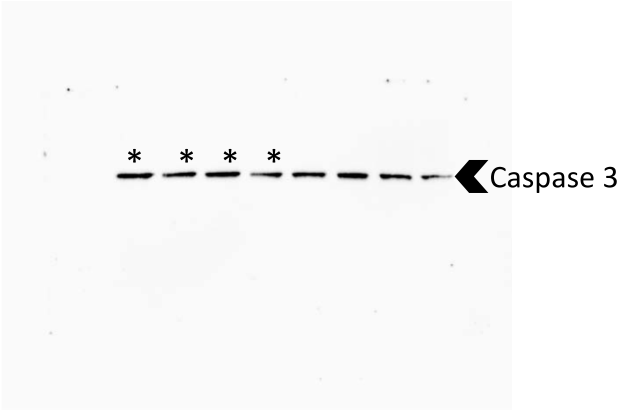

Fig 5C

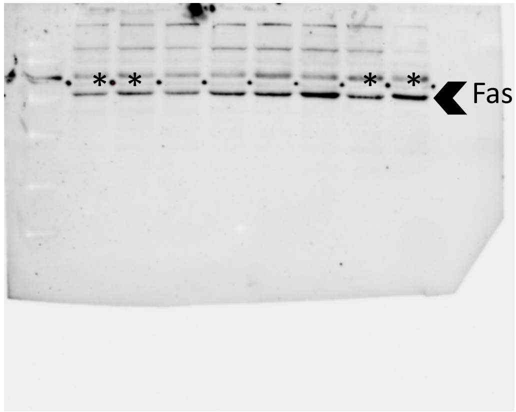

Fig 5C

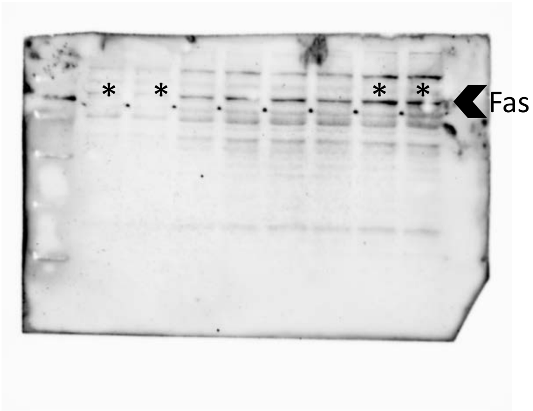

Fig 5D

Supplementary Figure S7

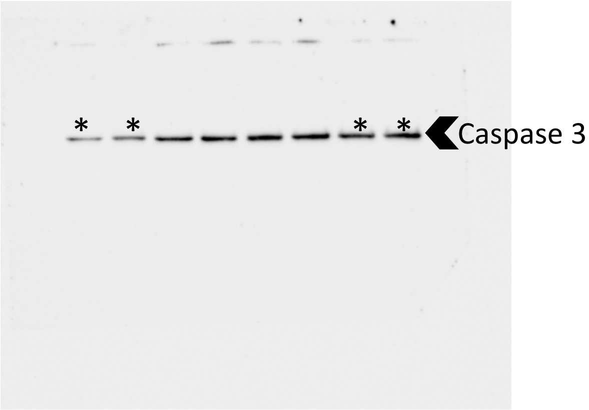

Fig 5D

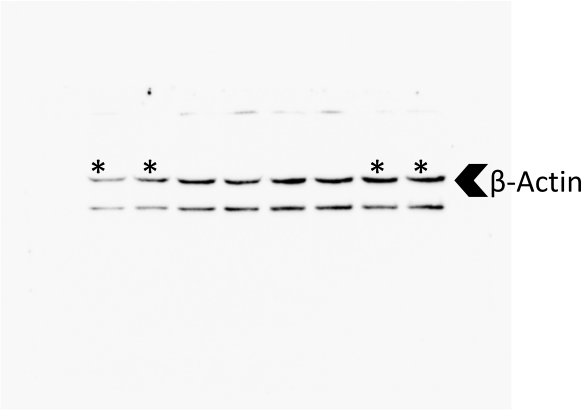

Fig 5D

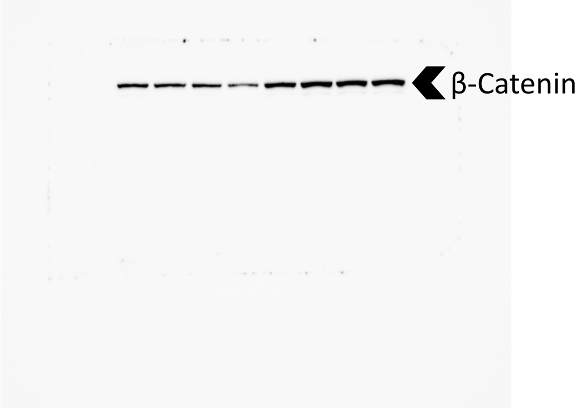

Fig 5 E & Fig 5 F

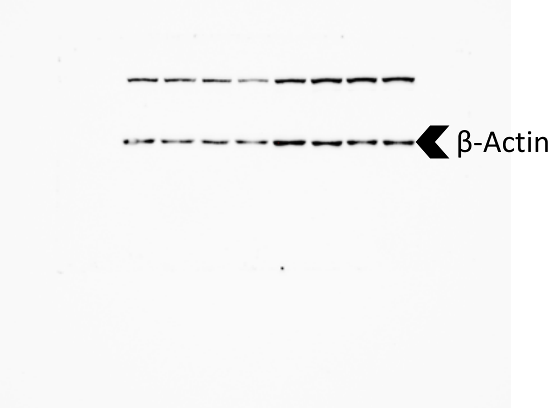

Fig 5 E & Fig 5 F

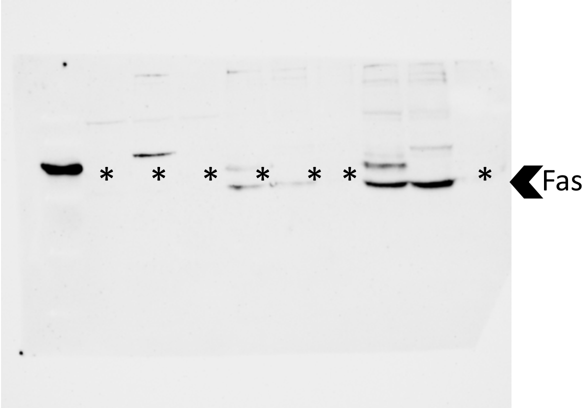

Fig S1

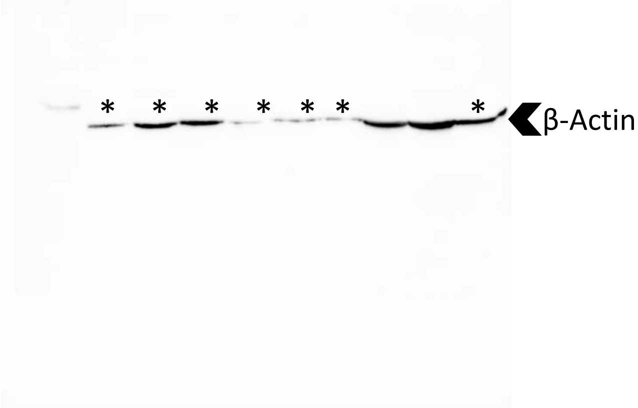

Fig S1

Supplementary Figure S8

Table S1. Fold changes of *UBC, UBB, UBA52* and *RPS27A* expression levels in 23132/87 and MKN45 gastric cancer cells, after normalization with GAPDH mRNA levels.

FOLD-CHANGES CALCULATED WITH GAPDH AS HOUSEKEEPING GENE

	UBC		UBB		UBA52		RPS27A	
	UBC 2^-ddCt		UBB 2^-ddCt		UBA52 2^-ddCt		RPS27A 2^-ddCt	
	mean	SD	mean	SD	mean	SD	mean	SD
23132/87	1,00	0,00	1,00	0,00	1,00	0,00	1,00	0,00
MKN45	1,66	0,54	1,16	0,11	1,02	0,19	1,69	0,26

Materials and Methods

Real-Time quantitative Polymerase Chain Reaction (RT-qPCR)

For gene-specific expression analysis, total RNA was isolated using the RNeasy Plus Mini kit (Qiagen, Hilden, Germany). 0.5 µg of total RNA were reverse transcribed using Primescript RT Master Mix (Perfect Real Time; Takara Bio Europe SAS, Saint-Germain-en-Laye, France) with oligo-dT and random hexamer primers, following the manufacturer’s instructions. qPCR detection and expression analysis of genes was performed with SYBR green quantitative real-time PCR, using Hot-Rescue Real Time PCR Kit (Diatheva s.r.l., Cartoceto PU, Italy), according to the manufacturer’s instructions. Briefly, the reaction was set up in a 25 µl final volume, using 5 ng cDNA as template and 200 nM of each specific primer. For RT-qPCR amplifications, 40 PCR cycles were run with the following thermal profile: 15 s at 95 °C melting temperature, 15 s at 60 °C annealing and 1 min at 72 °C extension temperature per cycle; before cycling, 10 min at 95 °C were allowed for Hot-Rescue Taq DNA polymerase activation. Fluorescence intensity of each amplified sample was measured with an ABI PRISM 7700 Sequence detection system (Applied Biosystems, Foster City, CA, USA). All measurements were performed at least in triplicate and reported as the average values ± standard deviation of the mean (mean ± SD). Target gene values were normalized with **GAPDH** mRNA measurements and expression data were calculated according to the $2^{-\Delta\Delta Ct}$ method [Livak, K.J.; Schmittgen, T.D. Analysis of relative gene expression data using real-time quantitative PCR and the 2(-Delta Delta C(T)) Method. *Methods* **2001**, *25*, 402-408].

Table S2. Fold changes of C-MYC expression levels in 23132/87 and MKN45 gastric cancer cells, after YY1 silencing.

	23132/87			MKN45	
	Mean	SD		Mean	SD
siGFP	1.00	0.00	siGFP	1.00	0.00
siYY1	0.72	0.01	siYY1	0.48	0.05
	**			**	

C-MYC mRNA was measured by RT-qPCR, in both 23132/87 and MKN45 cells transfected with siYY1 and siGFP as a control. The mRNA levels of c-MYC were normalized to B2M levels and expressed as fold change relative to siGFP transfected cells set as 1. Data shown are the means ± SD of three independent experiments. Asterisks denote statistical significance; **p< 0.01.

Materials and Methods

Real-Time quantitative Polymerase Chain Reaction (RT-qPCR)

For gene-specific expression analysis, total RNA was isolated using the RNeasy Plus Mini kit (Qiagen, Hilden, Germany). 0.5 µg of total RNA were reverse transcribed using Primescript RT Master Mix (Perfect Real Time; Takara Bio Europe SAS, Saint-Germain-en-Laye, France) with oligo-dT and random hexamer primers, following the manufacturer’s instructions. qPCR detection and expression analysis of genes was performed with SYBR green quantitative real-time PCR, using Hot-Rescue Real Time PCR Kit (Diatheva s.r.l., Cartoceto PU, Italy), according to the manufacturer’s instructions. Briefly, the reaction was set up in a 25 µl final volume, using 5 ng cDNA as template and 200 nM of each specific primer. For RT-qPCR amplifications, 40 PCR cycles were run with the following thermal profile: 15 s at 95 °C melting temperature, 15 s at 60 °C annealing and 1 min at 72 °C extension temperature per cycle; before cycling, 10 min at 95 °C were allowed for Hot-Rescue Taq DNA polymerase activation. Fluorescence intensity of each amplified sample was measured with an ABI PRISM 7700 Sequence detection system (Applied Biosystems, Foster City, CA, USA). All measurements were performed at least in triplicate and reported as the average values ± standard deviation of the mean (mean ± SD). Target gene values were normalized with **B2M** mRNA measurements and expression data were calculated according to the $2^{-\Delta\Delta Ct}$ method [Livak, K.J.; Schmittgen, T.D. Analysis of relative gene expression data using real-time quantitative PCR and the 2(-Delta Delta C(T)) Method. *Methods* **2001**, *25*, 402-408].

Supplementary Table S3.

Quantification of free Ub content in 23132/87 and MKN45 gastric cancer cells, after *UBB*, *UBC* and *UBB+UBC* silencing.

23132/87 (ng free Ub/μg protein)				MKN45 (ng free Ub/μg protein)			
	Mean	SD	P		Mean	SD	P value
siGFP	1.29	0.309		siGFP	1.11	0.169	
siUBB	0.77	0.130	**	siUBB	0.66	0.091	**
siUBC	0.79	0.125	**	siUBC	0.86	0.068	ns
siUBB+siUBC	0.64	0.107	**	siUBB+siUBC	0.62	0.142	**

Absolute quantification of free Ub (n=2) using 5 μg of protein extracts of GC cells, transfected with siRNAs as indicated, and a linear range of Ub standards run in parallel (for details see Figure S2). Results are given as ng free Ub/μg protein. Asterisks denote statistical significance versus the siGFP control; **$P < 0.01$; ns, not significant.

Materials and Methods

Western blot analysis

Proteins were resolved by SDS polyacrylamide gel electrophoresis (SDS PAGE) and electroblotted onto a nitrocellulose membrane (0.2 μm pore size) (Bio-Rad). The blots were probed with the following primary antibody: anti-Ub (rabbit polyclonal, kindly provided by Prof. A. L. Haas, Louisiana State University, Health Sciences Center, New Orleans) . Immunoreactive bands were detected by horseradish peroxidase (HRP)-conjugated secondary antibodies (Bio-Rad). Peroxidase activity was detected with the enhanced chemiluminescence detection method (WesternBright ECL, Advasta, Menlo Park, CA, USA) using ChemiDoc MP Imaging System (Bio-Rad).

Free ubiquitin quantification

To quantify free Ub protein levels, whole extracts (5 µg) were loaded and run in parallel with different amounts of purified ubiquitin (SIGMA Aldrich) used as reference standard, and submitted to western immunoblotting analysis with an antibody against Ub [53]. The adjusted volume intensity of the ubiquitin immunoreactive bands, in both Ub standard and cell sample lanes, was determined using Image Lab analysis software version 5.2.1. Calibration curves were generated, for each immunoblot, by plotting band intensities (adjusted volumes) against Ub standard concentrations. A regression line equation was then generated and used to calculate the free Ub concentration in the cell protein samples. The coefficient of determination (R^2) for ubiquitin standard curves was always between 0.98-0.99.
